# Pharmacological interventions for preventing opioid-induced hyperalgesia in adults after opioid-based anesthesia: a systematic review and network meta-analysis

**DOI:** 10.3389/fphar.2023.1199794

**Published:** 2023-06-22

**Authors:** Wei-Ji Xie, Ji-Shuang Hong, Cheng-Fei Feng, Hao-Feng Chen, Wei Li, Yong-Chun Li

**Affiliations:** ^1^ Department of Anesthesiology, State Key Laboratory of Oncology in Southern China, Sun Yat-sen University Cancer Center, Collaborative Innovation Center for Cancer Medicine, Guangzhou, China; ^2^ Department of Anesthesiology, The First Affiliated Hospital, Sun Yat-sen University, Guangzhou, China

**Keywords:** opioid-induced hyperalgesia, pharmacological interventions, general anesthesia, network meta-analysis, postoperative pain, postoperative nausea and vomiting

## Abstract

**Background:** Opioid-induced hyperalgesia (OIH) is an adverse event of prolonged opioid use that increases pain intensity. The optimal drug to prevent these adverse effects is still unknown. We aimed to conduct a network meta-analysis to compare different pharmacological interventions for preventing the increase in postoperative pain intensity caused by OIH.

**Methods:** Several databases were searched independently for randomized controlled trials (RCTs) comparing various pharmacological interventions to prevent OIH. The primary outcomes were postoperative pain intensity at rest after 24 h and the incidence of postoperative nausea and vomiting (PONV). Secondary outcomes included pain threshold at 24 h after surgery, total morphine consumption over 24 h, time to first postoperative analgesic requirement, and shivering incidence.

**Results:** In total, 33 RCTs with 1711 patients were identified. In terms of postoperative pain intensity, amantadine, magnesium sulphate, pregabalin, dexmedetomidine, ibuprofen, flurbiprofen plus dexmedetomidine, parecoxib, parecoxib plus dexmedetomidine, and S (+)-ketamine plus methadone were all associated with milder pain intensity than placebo, with amantadine being the most effective (SUCRA values = 96.2). Regarding PONV incidence, intervention with dexmedetomidine or flurbiprofen plus dexmedetomidine resulted in a lower incidence than placebo, with dexmedetomidine showing the best result (SUCRA values = 90.3).

**Conclusion:** Amantadine was identified as the best in controlling postoperative pain intensity and non-inferior to placebo in the incidence of PONV. Dexmedetomidine was the only intervention that outperformed placebo in all indicators.

**Clinical Trial Registration:** https://www.crd.york.ac. uk/prospero/display_record.php?, CRD42021225361.

## What is already known about this subject?

OIH is highly prevalent in surgery patients, contributing to various undesirable outcomes, such as more severe postoperative pain, increased opioid demand, and a high incidence of side effects.

In clinical routine, multiple medications with various mechanisms were proven to prevent the increase in postoperative pain caused by OIH. However, the comparative effects of different pharmacological interventions are urgently needed for a better guideline for the individualized anesthesia protocols.

### What this study adds

Amantadine, magnesium sulphate, pregabalin, dexmedetomidine, ibuprofen, flurbiprofen plus dexmedetomidine, parecoxib, parecoxib plus dexmedetomidine, and S (+)-ketamine plus methadone all have statistically significantly lower pain intensity than placebo. Amantadine was the most effective compared to placebo but failed to demonstrate superiority in the incidence of PONV.

Dexmedetomidine is not the best option, but it is the most well-balanced choice because it is the only intervention that outperforms placebo in all indicators.

## 1 Introduction

Opioids are the most commonly used analgesics during the perioperative period as a part of balanced anesthesia. Timely opioid administration during surgery reduces the need for general anesthetics, resulting in faster recovery (Lang et al., 1996), and post-surgery patient-controlled opioid analgesia improves patient comfort and satisfaction (McNicol et al., 2015). However, a state of nociceptive sensitization with reduction in nociceptive thresholds and paradoxical increase in pain after exposure to opioids (Koppert et al., 2003), referred to as opioid-induced hyperalgesia (OIH), have been demonstrated in animal models (Minville et al., 2010), human volunteers (Vinik and Kissin, 1998) and surgical patients (Fletcher and Martinez, 2014). Patients who experience more severe postoperative pain due to nociceptive sensitization may be obliged to accept more opioids unless alternatives are considered (He et al., 2020). Furthermore, opioid-related adverse drug events have been associated with increased inpatient mortality, prolonged stay, and a high cost of hospitalization (Shafi et al., 2018).

Although the precise molecular mechanism underlying OIH is unknown, it is widely assumed to be triggered by neuroplastic changes in the peripheral and central nervous systems (Lee et al., 2011). Previous research has demonstrated that opioids contribute to the occurrence of OIH by inhibiting glutamate recapture and inducing production of pro-inflammatory molecules (Roeckel et al., 2016). The inhibition of the glutamate transporter leads to increased synaptic concentrations of glutamate, which, in turn, activates the N-methyl-D-aspartate (NMDA) receptor, thus triggering OIH (Antal et al., 2008; Arout et al., 2015). Previous electrophysiological studies also identified the rapid and persistent upregulation of NMDA receptor function by clinically relevant concentrations of remifentanil, mirroring the potential target for the pathologic activation of NMDA receptor in the intervention of OIH (Guntz et al., 2005; Zhao and Joo Daisy, 2008). Furthermore, neuroinflammation mediated by opioid-triggered release of pro-inflammatory molecules and the activation of glial cells can sensitize pain pathways, lower pain thresholds, and contribute to the development of OIH (Grace et al., 2015). Additionally, the interactions between mu and delta opioid receptors (Beaudry et al., 2015),α-2 adrenoreceptors (Mercieri et al., 2017), neurokinin-1 receptor mediated transmission (Vera-Portocarrero et al., 2007), and spinal dynorphin expression (Vanderah et al., 2001) also have been reported to play a role in the development and maintenance of OIH.

In light of these findings, clinical investigators mainly focused on manipulating the glutaminergic system through modulation of the NMDA receptor and blocking the neuroinflammation to prevent the occurrence and development of OIH. Various interventions have been explored, including NMDA receptor antagonists (amantadine (Snijdelaar Dirk et al., 2004), magnesium sulphate (Ryu et al., 2008), methadone (Tognoli et al., 2020), and ketamine (Leal et al., 2015)), non-steroidal anti-inflammatory drugs (NSAIDs) (Ibuprofen (Koo et al., 2016), flurbiprofen (Zhang et al., 2016), and parecoxib (Du et al., 2019)), opioid receptor antagonist (naloxone (Du et al., 2019)), agonist-antagonist opioid analgesics (nalbuphine (Hu et al., 2020) and buprenorphine (Mercieri et al., 2017)) and α-2 adrenoceptor agonist (dexmedetomidine (Wu et al., 2021)). These interventions, with different mechanisms of action, have been shown the potential in reduce pain intensity and the need for postoperative analgesics due to OIH. Regretfully, clinical routines are still debatable about the optimal intervention strategy to prevent the increase in postoperative pain intensity caused by OIH due to small sample sizes and varying medication dosages in existing literature (Wu et al., 2015). Importantly, the relative effects of different types of medications remain unknown.

Given these uncertainties, we conducted a systematic review and network meta-analysis of various pharmacological interventions to prevent the increase in postoperative pain intensity caused by OIH in adults following general anesthesia, hoping better to guide clinical practice for more individualized general anesthesia protocols.

## 2 Methods

This network meta-analysis was registered on https://www.crd.york.ac.uk/PROSPERO. The registration number is CRD42021225361.

### 2.1 Search strategy and selection criteria

According to PRISMA Extension Statement for Reporting of Systematic Reviews Incorporating Network Meta-analyses (Hutton et al., 2015), MEDLINE, Embase, The Cochrane Central Register of Controlled Trials, and Web of Science were searched in English with language restrictions. The search strategy combined free text words and medical subject heading (MeSH) terms to maximize the results. The following keywords were used in the search: 1) opioid, 2) hyperalgesia, and 3) magnesium, naloxone, buprenorphine, ketamine, dexmedetomidine, butorphanol, propofol, flurbiprofen, morphine, methadone, lornoxicam, nitrous oxide, parecoxib, clonidine, amantadine, nalbuphine, paracetamol, pregabalin, nefopam, acetazolamide.

### 2.2 Selection of studies and data extraction

Two investigators (WJX and HFC) reviewed all titles, abstracts, and full texts sequentially. Finally, eligible trials were identified, and data on eligibility, quality, and outcomes were independently retrieved. Disagreements between the two reviewers on eligibility were resolved through mutual discussion. A third reviewer (YCL) was requested for the final decision when needed. Relevant data were extracted from eligible literature using a standard extraction formula and cross-checked.

Retrieved data included: 1) first author, year of publication, study location, study design, sample size, gender, age, American Society of Anesthesiologists (ASA) status, types of surgery, premedication, anesthesia maintenance, intervention description, control description, dose of opioid, postoperative analgesic strategies, and 2) pain intensity in the form of the various pain scores during the 0 to 24 postoperative hours, pain threshold or normalized area of hyperalgesia during the 0 to 48 postoperative hours, cumulative morphine consumption at 24 h after surgery, time to first rescue analgesic, and incidence of postoperative opioid-related side-effects, such as postoperative nausea and vomiting (PONV), shivering, dizziness and hypotension. Dichotomous data were extracted as the number of patients (%). Continuous data were extracted in the form of mean ± standard deviation (SD).

We tried to contact the author via e-mail twice when the target data in the article were incomplete, but no responses were received. Range and median estimation (Grace et al., 2015) were used to convert the data when the standard deviation was missing.

### 2.3 Type of outcome measures

Our primary outcomes were postoperative pain intensity at rest after 24 h and the incidence of PONV. Postoperative pain intensity was measured using pain scores scaling from 0 (no pain) to 10 (worst possible pain). Intensity scores reported on a visual analogue scale (VAS: 0: no pain to 100: worst possible pain) were transformed to a 0–10 scale. PONV, the most common adverse event, contributes to the highly distressing experience and severe patient dissatisfaction (Myles et al., 2000; Eberhart et al., 2002), with an incidence as high as 80% in high-risk cohorts (Apfel Christian et al., 1999).

Secondary outcomes include pain thresholds at 24 h after surgery, cumulative morphine consumption over the 24 h period, time to first postoperative analgesic requirement, and shivering incidence.

### 2.4 Assessment of risk of bias

Two investigators (WJX and HFC) read the eligible articles independently. They assessed their methodological validity using the Cochrane Collaboration’s tool of Review Manager software (RevMan version 5.4, Cochrane Community, London, England) for evaluating the risk of bias in randomized controlled trials (RCTs), and disagreements were resolved through discussion (Higgins et al., 2011). The tool includes seven items that describe random sequence generation, allocation concealment, participants and personnel blinding, outcome assessment, blinding, incomplete outcome data, selective reporting, and other biases. Each item was assigned a risk of material bias judgment of high, low, or unclear.

### 2.5 Statistical analysis

For dichotomous outcomes, odds ratios (ORs) with 95% confidence intervals (CIs) were calculated, as were standardized mean differences (SMDs) or mean differences (MDs) with 95% CIs for continuous outcomes.

This network meta-analysis was performed within a frequentist framework using the STATA 16.0 (StataCorp, Texas, United States) command ‘mvmeta’ (White, 2011). First, a network geometry plot for each outcome was created, which provided a visual and concise description of the relationship between pairs of interventions (Chaimani et al., 2013). Second, the node-splitting method and loop inconsistency mode were used to assess the statistical consistency. *p*-value ≥0.05 or 95% CI for each closed-loop containing 0 means direct and indirect comparisons were considered consistent (van Valkenhoef et al., 2016). Third, a comparison-adjusted funnel plot was used to assess publication bias. A symmetrical graph indicated that publication bias had a low influence, whereas an asymmetric graph indicated possible publication bias. Finally, the forest plot was used to report the results of the mixed comparison of interventions and placebo, and the league table was used to illustrate all head-to-head comparisons. We assumed that 95% of CIs that did not contain 0 were statistically significant for SMDs or MDs, and those that did not have 1 were statistically significant for ORs. The two-dimensional graph is prepared to visualize comprehensive drug for placebo comparisons. The point in the lower-left portion of the coordinate system that does not intersect with the dark grey dashed line indicates that this pharmacological intervention outperforms the placebo regarding postoperative pain intensity and PONV incidence. Furthermore, the ranking probabilities of all interventions at each possible intervention rank were estimated (Chaimani et al., 2013). The treatment hierarchy was summarized and reported as the surface under the cumulative ranking curve (SUCRA) (Chaimani et al., 2013) the ranking probabilities. The higher the SUCRA value, the higher the rank of the treatment outcomes.

### 2.6 Inclusion and exclusion criteria

RCTs that met the following criteria were considered eligible: 1) anesthesia was induced and maintained with opioids; 2) pharmacological interventions were administered to patients at any dose before or during the operative period; and 3) pharmacological interventions were compared to the placebo.

Articles were excluded based on the following criteria: 1) combination with regional nerve block during the anesthesia induction or maintenance period, and 2) data from healthy volunteer or pediatric studies, abstracts, letters, or reviews.

## 3 Results

### 3.1 Study selection and characteristics

**FIGURE 1:**
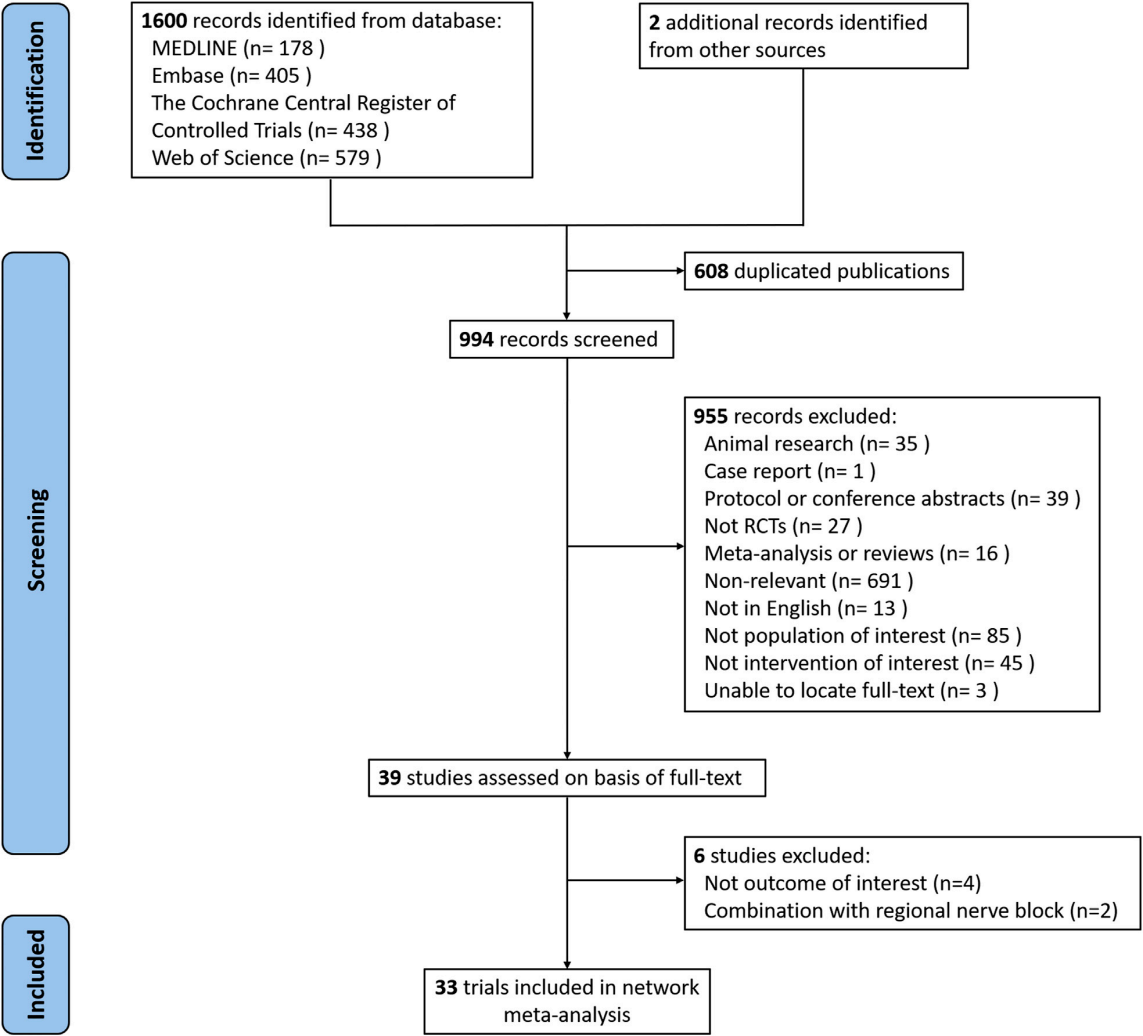
Flow chart of search strategy to identify the eligible randomized controlled trials (RCTs).

We identified 1,602 potentially relevant studies in total. After adjusting for duplicates and reviewing the title/abstract, the remaining 39 full-text manuscripts were reviewed. Following the study protocol, six trials were excluded due to a lack of outcome of interest (n = 4) and a combination with a regional nerve block (n = 2). In total, 33 RCTs with a total of 1711 patients were identified. Figure 1 depicts the process of literature selection.

A total of 960 subjects were randomly assigned to pharmacological intervention and 751 to placebo. The included RCTs were published between 2002 and 2020 and included orthopedic (n = 2), urinary (n = 4), abdominal (n = 10), gynecologic (n = 9), thyroid surgery (n = 5), thoracic (n = 1) and ear-nose-throat surgery (n = 2). Table 1 describes the basic characteristics of the enrolled studies.

**TABLE 1:** Characteristics of studies.

Study (author/year)	Country	Sample Size (intervention/control)	Gender (M/F)	Mean Age	ASA Status (Ⅰ/Ⅱ/Ⅲ)	Intervention	Type of Surgery	Does of Opioid
W. Jaksch 2002	Austria	15/15	15/15	31.5	NA	S (+)-ketamine 0.5 mg/kg IV + 2.0 µg/kg· min continuous infusion	Arthroscopic anterior cruciate ligament repair	Remifentanil 0.125–1.0 μg/kg· min
B. Guignard 2002	France	25/25	14/16	62.5	9/35/7	Ketamine 0.15 mg/kg IV + 2.0 µg/kg· min continuous infusion	Open colorectal surgery	Remifentanil 0.25 μg/kg· min
A. Sahin 2004	Turkey	17/16	16/17	47.4	NA	Ketamine 0.5 mg/kg IV	Lumbar disk operation	Remifentanil 0.1 μg/kg· min
A. C. Van Elstraete 2004	France	20/20	20/20	29.0	NA	Ketamine 0.5 mg/kg IV + 2.0 µg/kg· min continuous infusion	Elective electrodissection tonsillectomy	Remifentanil 0.125–1.0 μg/kg· min
D. G. Snijdelaar 2004	Canada	11/10	21/0	60.0	8/12/1	Amantadine 200 mg orally at night and at 1 h before surgery and 100 mg at 8, 20, and 32 h after surgery	Radical prostatectomy	NA
V. Joly 2005	France	24/25	18/32	57.5	21/22/7	Ketamine 0.5 mg/kg IV + 5.0 µg/kg· min continuous infusion +2.0 µg/kg· min for 48 h after surgery	Abdominal surgery	Remifentanil 0.4 μg/kg· min
J. H. Ryu 2008	Korea	25/25	0/50	42.4	37/13/0	Magnesium sulphate 50 mg/kg IV + 15 mg/kg· h continuous infusion	Total abdominal hysterectomy	Remifentanil TCI 4 ng/mL
S. Kaya 2009	Turkey	20/20	NA	50.0	NA	Magnesium sulphate 30 mg/kg IV + 500 mg/h continuous infusion	Elective abdominal hysterectomy	Remifentanil 0.25 μg/kg· min
H. R. Jo 2011	Korea	20/20	0/40	46.1	34/6/0	Pregabalin 150 mg orally	Non-malignant total abdominal hysterectomy	Remifentanil TCI 3–4 ng/mL
C. Lee 2011	Korea	25/25	50/0	63.4	NA	Magnesium sulfate 80 mg/kg IV	Robot-assisted laparoscopic prostatectomy	Remifentanil 0.3 μg/kg· min
C. Lee 2011	Korea	30/30	38/22	38.2	NA	Adenosine 80 µg/kg· min continuous infusion	Tonsillectomy	Remifentanil 0.1 μg/kg· min
J. W. Song 2011	Korea	28/28	11/45	46.0	NA	Magnesium sulphate 30 mg/kg IV + 10 mg/kg· h continuous infusion	Thyroidectomy	Remifentanil 0.2 μg/kg· min
H. Bornemann-Cimenti 2012	Germany	13/13	11/15	56.9	NA	Pregabalin 300 mg orally	Elective transperitoneal nephrectomy	Remifentanil 0.1–0.5 μg/kg· min
C. Lee 2013	Korea	28/29	0/57	48.7	NA	Dexmedetomidine 1.0 µg/kg IV + 0.7 µg/kg· h continuous infusion	Laparoscopically assisted vaginal hysterectomy	Remifentanil 0.3 μg/kg· min
C. Lee 2013	Korea	31/29	31/29	50.7	NA	Pregabalin 300 mg orally	Laparoendoscopic single-site urologic surgery	Remifentanil 0.3 μg/kg· min
S. Treskatsch 2014	Germany	16/17	8/25	66.0	NA	Amantadine 200 mg/500 mL solution	Intra-abdominal surgery	Remifentanil 0.2 μg/kg· min
E. Choi 2015	Korea	25/25	0/50	44.1	NA	Ketamine 0.5 mg/kg IV + 5.0 µg/kg· min continuous infusion	Elective laparoscopic gynecological surgery	Remifentanil 0.3 μg/kg· min
P. C. Leal 2015	Brazil	28/28	9/47	44.6	28/28/0	Ketamine 5.0 µg/kg· min continuous infusion	Laparoscopic cholecystectomy	Remifentanil 0.4 μg/kg· min
H. Bornemann-Cimenti 2016	Austria	37/19	31/25	60.5	4/24/28	S (+)-ketamine 0.25 mg/kg IV + 0.125 mg/kg· h continuous infusion or S (+)-ketamine 0.015 mg/kg· h continuous infusion	Elective major abdominal surgery	Remifentanil 0.1–0.3 μg/kg· min
M. Kong 2016	China	25/25	32/18	51.5	NA	Butorphanol 0.2 µg/kg IV + 0.02 µg/kg· min continuous infusion	Laparoscopic cholecystectomy	Remifentanil 0.3 μg/kg· min
C.-H. Koo 2016	Korea	27/26	33/20	63.7	NA	Ibuprofen 800 mg IV over 30 min	Pancreaticoduodenectomy	Remifentanil TCI 4 ng/mL
Z. Yu 2016	China	57/29	0/86	46.1	NA	Dexmedetomidine 0.5 µg/kg IV + 0.6 µg/kg h continuous infusion or flurbiprofen 1.5 mg/kg combination with dexmedetomidine infusion	Laparoscopic assisted vaginal hysterectomy	Remifentanil 0.3 μg/kg· min
L. Zhang 2016	China	56/28	0/84	46.0	67/NA/NA	Butorphanol 20 µg/kg IV or butorphanol 20 µg/kg combined with flurbiprofen 0.5 mg/kg	Elective laparoscopicgynaecological surgery	Remifentanil 0.3 μg/kg· min
C. H. Koo 2017	Korea	30/31	20/41	47.0	50/11/0	Naloxone 0.05 µg/kg· min continuous infusion	Thyroid surgery	Remifentanil TCI 4 ng/mL
M. Mercieri 2017	Italy	31/32	34/29	64.5	6/42/15	Buprenorphine 25 µg/h continuous infusion	Lateral thoracotomy	Remifentanil TCI 5 ng/mL
L. Zhang 2017	China	28/28	0/56	44.8	45/11/0	Flurbiprofen 1.0 mg/kg IV	Elective laparoscopic gynecologic surgery	Remifentanil 0.3 μg/kg· min
H. Qiu 2018	China	32/16	24/24	NA	NA	Dexmedetomidine 0.2 µg/kg IV or 0.6 µg/kg	Thyroidectomy	Remifentanil 0.2 μg/kg· min
B. Sng 2018	Singapore	45/44	0/89	48.1	NA	S (+)-ketamine 0.25 mg/kg IV	Open abdominal hysterectomy	NA
X. Du 2019	China	60/20	NA	NA	NA	Parecoxib 40 mg IV or dexmedetomidine 0.6 µg/kg· h continuous infusion or both	Laparoscopic cholecystectomy	NA
R. Gutiérrez 2019	Chile	23/24	4/43	44.5	18/29/0	ACTZ 250 mg IV	Total thyroidectomy without neck dissection	Remifentanil TCI 4.5 ± 0.5 ng/mL
J. Hu 2020	China	24/24	11/37	50.2	24/24/0	Nalbuphine 0.2 mg/kg IV	Laparoscopic cholecystectomy	Remifentanil 0.4 μg/kg· min
E. Tognoli 2020	Italy	24/24	29/19	58.6	19/24/5	S (+)-ketamine 5.0, 2.5 and 2 µg/kg· min continuous infusion + methadone 2.0 mg IV	Open laparotomy for anterior resection of the rectum	Remifentanil TCI 5–7 ng/mL
Z. Wu 2020	China	60/29	28/61	40.0	74/15/0	Dexmedetomidine 0.2 µg/kg continuous infusion or 0.5 µg/kg	Thyroidectomy	Remifentanil 0.3 μg/kg· min

### 3.2 Risk of bias assessment

Supplementary Appendix S2 contains the details for the risk of bias assessment. The random sequence generation was specified in 24 trials (72.7%). Although 18 trials (54.5%) reported allocation concealment, one trial had a high risk of bias. Only one trial did not use blinding methods. Eight trials (24.2%) had selective reporting. No trials were found to be at high risk of bias due to incomplete outcome data and other bias. Overall, the included studies were of relatively high quality.

### 3.3 Network geometry of eligible comparisons

**FIGURE 2:**
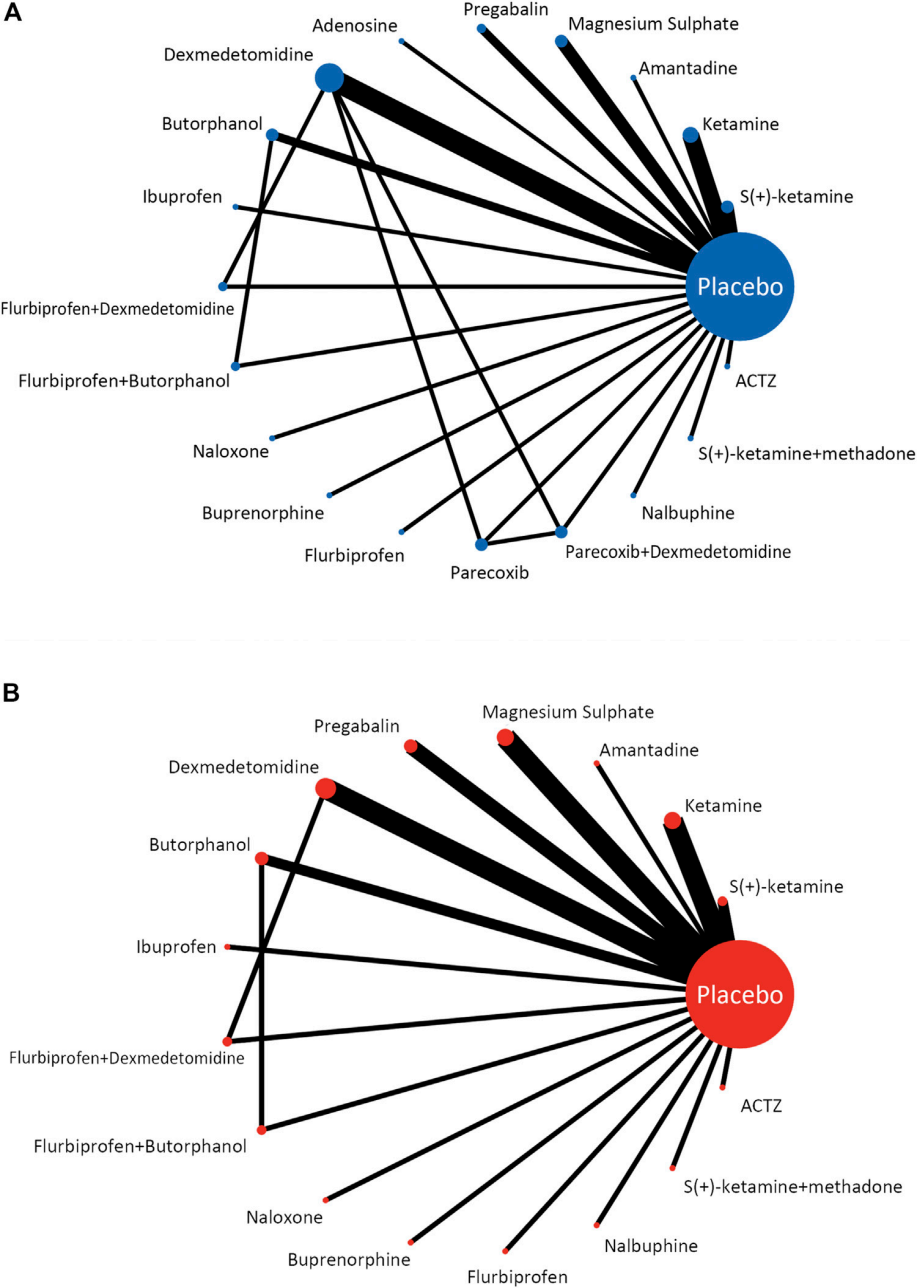
Network meta-analysis of eligible comparisons for postoperative pain intensity at rest at 24 h **(A)** and the incidence of PONV **(B)**.

The network geometry plot (Figure 2) represents the network of eligible comparisons for postoperative pain intensity at rest at 24 h (A) and the incidence of PONV (B). The postoperative pain intensity at rest at 24 h after surgery was reported in 28 studies involving 20 treatments, and the incidence of PONV was reported in 27 studies involving 17 treatments. There was at least one placebo-controlled trial for each treatment. When a direct comparison was performed, each treatment was represented by a node and linked by an edge. More sample sizes indicate a bigger node, while more studies demonstrate a thicker edge.

### 3.4 Results of primary outcomes

**FIGURE 3:**
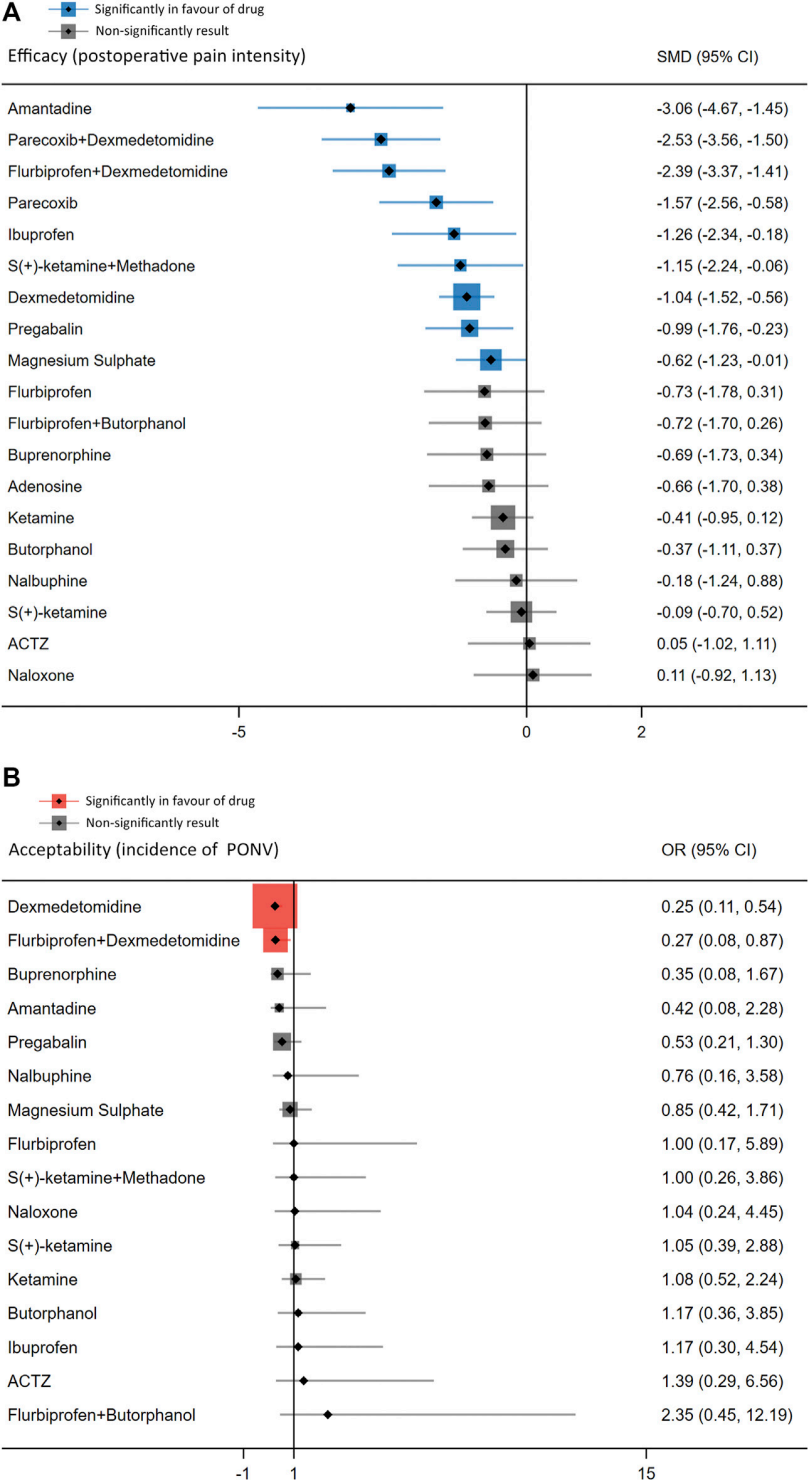
Forest plots of network meta-analysis of all trials for postoperative pain intensity at rest at 24 h **(A)** and the incidence of PONV **(B)**.

The forest plot (Figure 3) displays the network meta-analysis results for the primary outcomes. In terms of postoperative pain intensity, amantadine, magnesium sulphate, pregabalin, dexmedetomidine, ibuprofen, flurbiprofen plus dexmedetomidine, parecoxib, parecoxib plus dexmedetomidine and S (+)-ketamine plus methadone were all associated with milder pain intensity than placebo, with SMDs ranging between −3.06 (95% CI: −4.67, −1.45) for amantadine and −0.62 (95% CI: −1.23, −0.01) for magnesium sulphate. Regarding the PONV incidence, intervention with dexmedetomidine (OR = 0.25, 95% CI: 0.11, 0.54) or flurbiprofen plus dexmedetomidine (OR = 0.27, 95% CI: 0.08, 0.87) results in a lower incidence of PONV than placebo.

**FIGURE 4:**
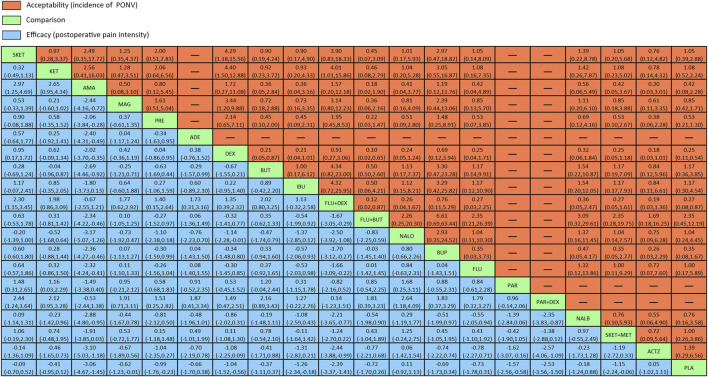
League table of head-to-head comparisons for postoperative pain intensity at rest at 24 h and the incidence of PONV of all pharmacological interventions and placebo. PLA = placebo. SKET = S (+)-ketamine. KET = ketamine. AMA = amantadine. MAG = magnesium sulphate. PRE = pregabalin. ADE = adenosine. DEX = dexmedetomidine. BUT = butorphanol. IBU = ibuprofen. FLU + DEX = flurbiprofen + dexmedetomidine. FLU + BUT = flurbiprofen + butorphanol. NALO = naloxone. BUP = buprenorphine. FLU = flurbiprofen. PAR = parecoxib. PAR + DEX = parecoxib + dexmedetomidine. NALB = nalbuphine. SKET + MET = S (+)-ketamine + methadone.

**FIGURE 5:**
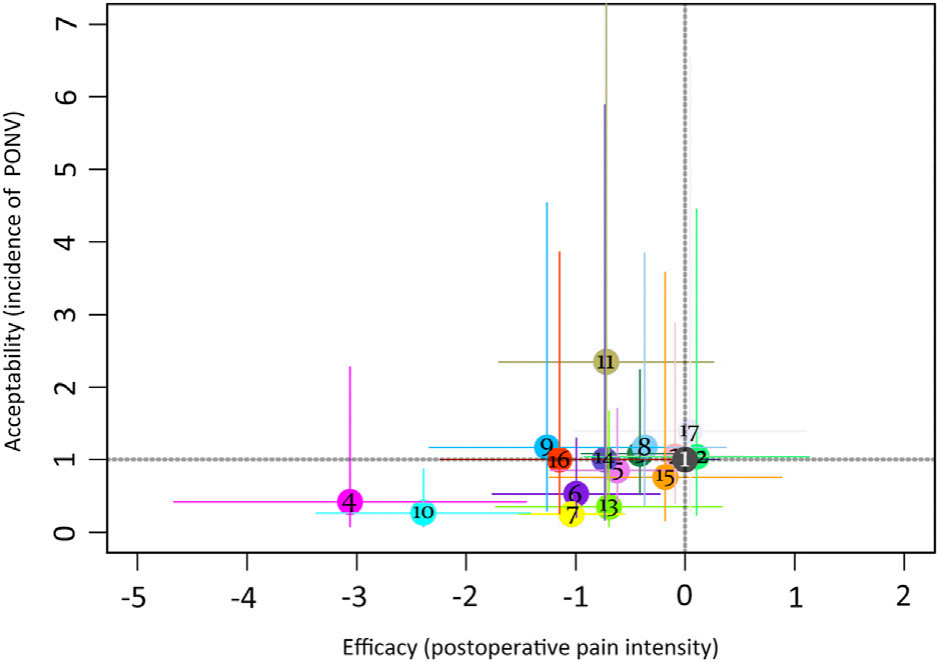
Two-dimensional graphs for postoperative pain intensity at rest at 24 h and the incidence of PONV. 1 = placebo; 2 = S (+)-ketamine; 3 = ketamine; 4 = amantadine; 5 = magnesium sulphate; 6 = pregabalin; 7 = dexmedetomidine; 8 = butorphanol; 9 = ibuprofen; 10 = flurbiprofen + dexmedetomidine; 11 = flurbiprofen + butorphanol; 12 = naloxone; 13 = buprenorphine; 14 = flurbiprofen; 15 = nalbuphine; 16 = S (+)-ketamine + methadone; 17 = ACTZ.

The league table (Figure 4) illustrates head-to-head comparisons of all pharmacological intervention strategies and placebo for postoperative pain intensity (lower left portion) and PONV incidence (upper right portion). The results of pairwise comparisons are expressed as SMD (95% CI) and OR (95% CI), respectively. The two-dimensional graph (Figure 5) reveals that only dexmedetomidine and flurbiprofen plus dexmedetomidine outperform placebo in terms of postoperative pain intensity and PONV incidence.

In the ranking probability plot (Supplementary Appendix S4, Figure 4), amantadine appeared to be the best agent for postoperative pain intensity among all 20 treatments with a SUCRA value of 96.2. In terms of PONV incidence, it was determined that dexmedetomidine appeared to be the best option among all 17 PONV treatments, with a SUCRA value of 90.3.

### 3.5 Results of secondary outcomes

#### 3.5.1 Pain threshold at 24 h after surgery

Ten studies involving 11 interventions reported pain thresholds 24 h after surgery (measured by QST and in g) (Supplementary Appendix S4 and Supplementary Figure 4.1.1). Butorphanol (SMD = 2.43, 95% CI: 1.65, 3.22), magnesium sulphate (SMD = 1.01, 95% CI: 0.14, 1.88) and dexmedetomidine (SMD = 1.01, 95% CI: 0.14, 1.88) have higher pain thresholds than placebo at 24 h after surgery (Supplementary Appendix S4 and Supplementary Figure 4.1.2). The league table (Supplementary Appendix S4 and Supplementary Figure 4.1.3) illustrates the comparison of each intervention to one another. Flurbiprofen plus dexmedetomidine was ranked first in the ranking probability plot (Supplementary Appendix S4 and Supplementary Figure 4.1.4) among 11 interventions with a SUCRA value of 98.1.

#### 3.5.2 Cumulative morphine consumption over the 24 h

A total of 14 studies with 11 interventions reported cumulative morphine consumption over a 24 h period (Supplementary Appendix S4 and Supplementary Figure 4.2.1). Flurbiprofen (SMD = −17.36, 95% CI: −22.13, −12.59) and dexmedetomidine (SMD = −11.83, 95% CI: −17.77, −5.90) caused more morphine consumption at 24 h after surgery than placebo (Supplementary Appendix S4 and Supplementary Figure 4.2.2). The league table (Supplementary Appendix S4 and Supplementary Figure 4.2.3) compares the outcomes of each intervention to one another. Flurbiprofen plus dexmedetomidine was ranked first in the ranking probability plot (Supplementary Appendix S4 and Supplementary Figure 4.2.4) among 11 interventions with a SUCRA value of 100.

#### 3.5.3 The time to first postoperative analgesic requirement

The time to the first postoperative analgesic requirement was reported in 14 studies involving 13 interventions (Supplementary Appendix S4 and Supplementary Figure 4.3.1). When compared with placebo, flurbiprofen plus dexmedetomidine (MD = 43.05, 95% CI: 28.49, 57.60), adenosine (MD = 26.90, 95% CI: 11.98, 41.82), magnesium sulphate (MD = 23.29, 95% CI: 12.27, 34.30) and dexmedetomidine (MD = 11.39, 95% CI: 0.93, 21.84) have a longer time to require first postoperative analgesic (Supplementary Appendix S5 and Supplementary Figure 5.3.2). The league table (Supplementary Appendix S4 and Supplementary Figure 4.3.3) compares the outcome of each intervention to one another. Flurbiprofen plus dexmedetomidine was ranked first in the ranking probability plot (Supplementary Appendix S4 and Supplementary Figure 4.3.4) among 13 interventions with SUCRA value of 98.5.

#### 3.5.4 Incidence of shivering

Nine studies involving nine interventions reported the incidence of shivering (Supplementary Appendix S4 and Supplementary Figure 4.4.1). Dexmedetomidine (OR = 0.16, 95% CI: 0.06, 0.43), flurbiprofen plus dexmedetomidine (OR = 0.12, 95% CI: 0.03, 0.49), magnesium sulphate (OR = 0.07, 95% CI: 0.02, 0.36) and S (+) -ketamine (OR = 0.05, 95% CI: 0.00, 0.99) have a lower incidence of shivering than placebo (Supplementary Appendix S4 and Supplementary Figure 4.4.2). The league table (Supplementary Appendix S4 and Supplementary Figure 4.4.3) compares the outcomes of each intervention to one another. S (+) -ketamine was ranked highest in the ranking probability plot (Supplementary Appendix S4 and Supplementary Figure 4.4.4) among nine interventions with SUCRA value of 82.0.

## 4 Discussion

Because of the morbidity concealment, complex pathogenesis, and treatment uncertainty of OIH, the best strategy is to avoid it. This is the first systematic review and network meta-analysis to compare various pharmacological interventions and investigate the best strategy for preventing the increase in postoperative pain caused by OIH in adults following general anesthesia. The following aspects of the 20 treatments were compared and analyzed: pain intensity, opioid-related adverse effects, pain threshold, time first to rescue analgesia, and morphine consumption. We identified that no such perfect drug performs best in all indicators. This emphasizes the significance of individualized treatment selection and a multimodal approach.

Our findings reveal that amantadine, magnesium sulphate, pregabalin, dexmedetomidine, ibuprofen, flurbiprofen plus dexmedetomidine, parecoxib, parecoxib plus dexmedetomidine and S (+)-ketamine plus methadone all have the potential to prevent the increase in postoperative pain intensity, with amantadine appearing to be the best option among the 20 interventions studies. Although the mechanisms underlying OIH are not fully understood. Preclinical models implicate the glutaminergic system and pathological NMDA receptor activation in the development of central sensitization (Mao et al., 1994; Mao et al., 2002; Zhao et al., 2012). Amantadine, magnesium sulphate, methadone, and S (+)-ketamine are known to be the NMDA receptor’s antagonists, where its primary effects are thought to occur. Wu L et al. found that perioperative administration of NMDA receptor antagonists effectively reduced postoperative pain intensity and morphine consumption (Wu et al., 2015), without evident psychological effects. However, our findings suggest that amantadine may be the best option when either ketamine or S (+)-ketamine fails to show significant superiority in preventing the rise of postoperative pain intensity. A possible explanation for this discrepancy is that Wu L et al.'s conclusion requires extraordinary caution in interpretation due to high heterogeneity even after subgroup analysis. The studies involved were small (only 14 studies included 3 drugs which directly act on NMDA receptors), with possible overestimation of the risk of Type II statistical error. However, the effect of an intervention may be influenced to varying degrees by other factors in NMDA. Therefore, we suggest that future studies should consider confirming the findings of our meta-analysis.

Ibuprofen, flurbiprofen, and parecoxib are NSAIDs that have potent anti-inflammatory, analgesic, and antipyretic activities and are used globally. One of their primary mechanisms of action is the inhibition of cyclo-oxygenase (COX), an enzyme involved in the biosynthesis of prostaglandins and thromboxane (Bacchi et al., 2012). Prostaglandins have been demonstrated to modulate nociceptive processing (Baba et al., 2001) and stimulate the release of the excitatory amino acid glutamate in the dorsal horns of the spinal cord (O’Rielly Darren and Loomis Christopher, 2006). Moreover, COX constitutively expressed in the spinal cord and is activated in response to peripheral stimuli that cause pain, primarily through the involvement of NMDA and substance P signaling (Yaksh and Malmberg, 1993). As such, NSAIDs have been demonstrated to inhibit the heightened sensitivity to pain triggered by the activation of spinal NMDA and substance P receptors (Malmberg and Yaksh, 1992; Yaksh and Malmberg, 1993). Clinical studies or meta-analyses about the effect of COX inhibitors on OIH are still lacking, even though it has been proved in animal models (Li et al., 2018; Peng et al., 2019) and human volunteers (Koppert et al., 2004; Lenz et al., 2011).

It has been indicated that opioid-induced pronociceptive effects are caused by central and peripheral nervous system sensitization, similar to the mechanism of hyperalgesia associated with nerve injury (Mao et al., 1995). Pregabalin is a 3-substituted analogue of γ-aminobutyric acid used to treat neuropathic pain (Guay, 2005) with the side effects of dizziness and drowsiness. It has a similar structure and mechanism of action to gabapentin but has fewer side effects (Ben-Menachem, 2004). Pregabalin binds strongly to the α2δ-1 subunit of voltage-gated calcium channels. This binding impairs channel trafficking and reduces the release of various neurotransmitters, including glutamate, noradrenaline, and substance P (Bannister et al., 2011). These effects result in interactions with spino-bulbo-spinal loop-comprising projection neurons in the superficial dorsal horn and brainstem, leading to facilitation of 5-hydroxytryptamine3 receptor-mediated effects in pain modulation. It has been indicated that pregabalin reduce hyperalgesia and allodynia in human volunteers (Chizh et al., 2007) and rat models (Field et al., 1999). However, A J Lederer et al. reviewed the effects of pregabalin on OIH and concluded that, despite strong support by theoretical considerations, the recommendation as a clinical use still lacks clinical evidence (Lederer et al., 2011). Stoicia et al. reached a similar conclusion, stating that applying gabapentin in mitigating OIH still requires support from large-scale standardized patient studies (Stoicea et al., 2015).

Dexmedetomidine is a potent and highly selective α-2 adrenoceptor agonist with sympatholytic, sedative, amnestic, and analgesic properties (Khan et al., 1999). Its anti-hyperalgesia effects are closely associated with NMDA receptors. Animal studies reveal that dexmedetomidine modulates spinal cord NMDA receptor activation by suppressing tyrosine phosphorylation of NR2B in the superficial spinal cord, which was found to be upregulated during remifentanil-induced hyperalgesia (Zheng et al., 2012). Furthermore, another study has provided evidence supporting the prevention of OIH by dexmedetomidine through the regulation of spinal NMDA receptors, as well as the levels of protein kinase C (PKC) and calcium/calmodulin-dependent protein kinase II (CaMKII), both of which are involved in neuronal signaling (Yuan et al., 2017). Similarly, its anti-hyperalgesia effect in clinical practice requires further investigation.

The findings of the present meta-analysis also revealed that dexmedetomidine and flurbiprofen plus dexmedetomidine are interventions associated with a lower PONV incidence compared to placebo. It is worth noting that flurbiprofen alone has no superior effect. This appears to imply that dexmedetomidine plays a significant role in preventing PONV, consistent with previous studies (Le Bot et al., 2015; Jin et al., 2017; Grape et al., 2019). Jin S et al. (Jin et al., 2017) investigated the effect of dexmedetomidine on PONV in patients undergoing general anesthesia. They identified that dexmedetomidine (irrespective of administration mode) had a significantly lower incidence of PONV than placebo. It was thought that this additional antiemetic effect of α2 agonists might be explained by inhibiting catecholamines by parasympathetic tone, even though the biological basis remains unknown. Alternatively, dexmedetomidine may reduce intraoperative anesthetics and opioids, which have been considered risk factors for PONV (Gan et al., 2020).

The treatment risk/benefit ratio is an important consideration in clinical decision-making. Our findings revealed that, while there is the best option in every index, dexmedetomidine is the only pharmacological intervention that outperformed placebo in all indicators. In addition, the multifaceted benefit of dexmedetomidine in improving the quality of emergence from anesthesia (Aouad et al., 2019), reducing postoperative delirium incidence (Duan et al., 2018), enhancing recovery after surgery (Kaye et al., 2020) and providing organ-protective effects (Bao and Tang, 2020) has already been fully demonstrated and widely accepted. Despite the side effects of hypotension and bradycardia, it is difficult to deny that dexmedetomidine is an attractive anesthetic adjuvant (Weerink et al., 2017).

This network meta-analysis had several possible limitations. First, because multiple interventions were included in the analysis, several had data from only one study, resulting in a relatively small sample size, which could have led to possible bias and overestimation of the treatment effect. Second, some non-pharmacological interventions, such as gradual withdrawal of remifentanil (Comelon et al., 2016), opioid rotation (Mercadante and Arcuri, 2005) and combination with a regional nerve block (Rivat et al., 2013), were not included in the comparison. Third, it is important to acknowledge that despite conducting comprehensive literature research prior to designing the retrieval strategy and considering commonly used drugs in clinical anesthesia, there is a possibility that our study may have omitted other drugs that have been investigated for their effects on OIH. Finally, there was variation in gender, opioid dosage, timing, administration regimens, surgery duration, and anesthesia maintenance. These disparities limit the amount of data pooled in a meta-analysis, posing significant challenges in interpreting and applying the results.

Overall, this systematic review and network meta-analysis provides the most comprehensive summary of the comparative effect of various pharmacological interventions on improving the intensity of postoperative pain caused by OIH.

## 5 Conclusion

In summary, a meta-analysis of eligible RCTs identified that amantadine was the best at preventing an increase in postoperative pain and non-inferior to placebo in the incidence of PONV. In contrast, dexmedetomidine was the only intervention superior to placebo in all indicators.

## Data Availability

The original contributions presented in the study are included in the article/Supplementary Material, further inquiries can be directed to the corresponding authors.
