# Supplementary material for: Pharmacological interventions for preventing opioid-induced hyperalgesia in adults after opioid-based anesthesia: a systematic review and network meta-analysis
Source: Front Pharmacol. 2023 Jun 22;14:1199794. doi: 10.3389/fphar.2023.1199794 (PMC10324676; doi:10.3389/fphar.2023.1199794)
Supplement: Supplementary file 1 [file DataSheet1.docx]

**Supplementary Appendix Content**

**Appendix 1.** Risk of Bias Assessment

**Appendix 2.** Comparison-adjusted Funnel Plot

**Appendix 3.** SUCRA and cumulative probability plots for efficacy and acceptability

**Appendix 4.** Results of secondary outcomes

**Appendix 4.1.** Pain thresholds at 24h

**Appendix 4.2.** Cumulative morphine consumption over the 24h

**Appendix 4.3.** The time to first postoperative analgesic requirement.

**Appendix 4.4.** Incidence of Shivering

**Appendix 1**

**Risk of Bias assessment**


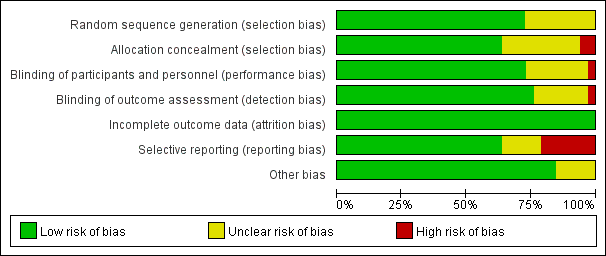


Figure 1.1: Risk of bias graph


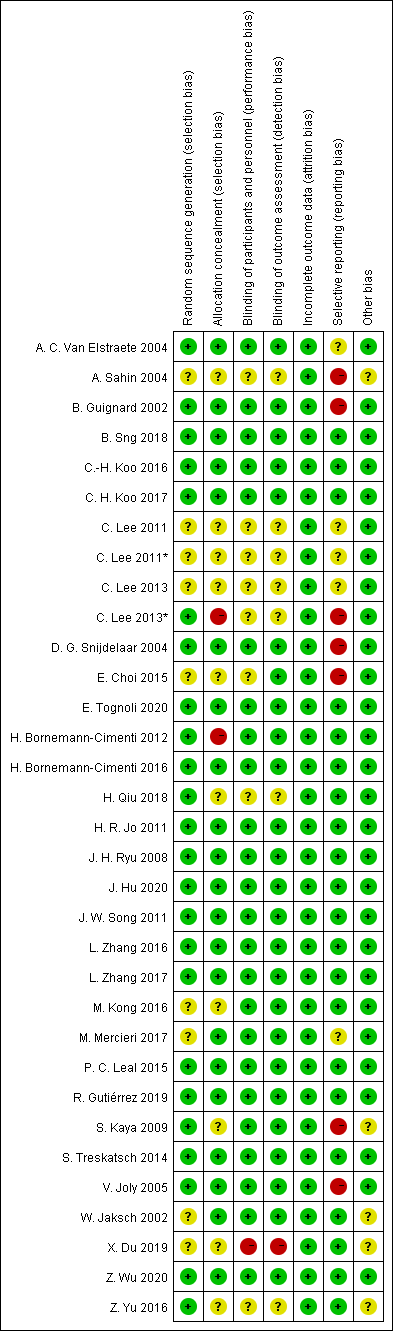


Figure 1.2: Risk of bias summary

**Appendix 2**

**Comparison-adjusted Funnel Plot**

**
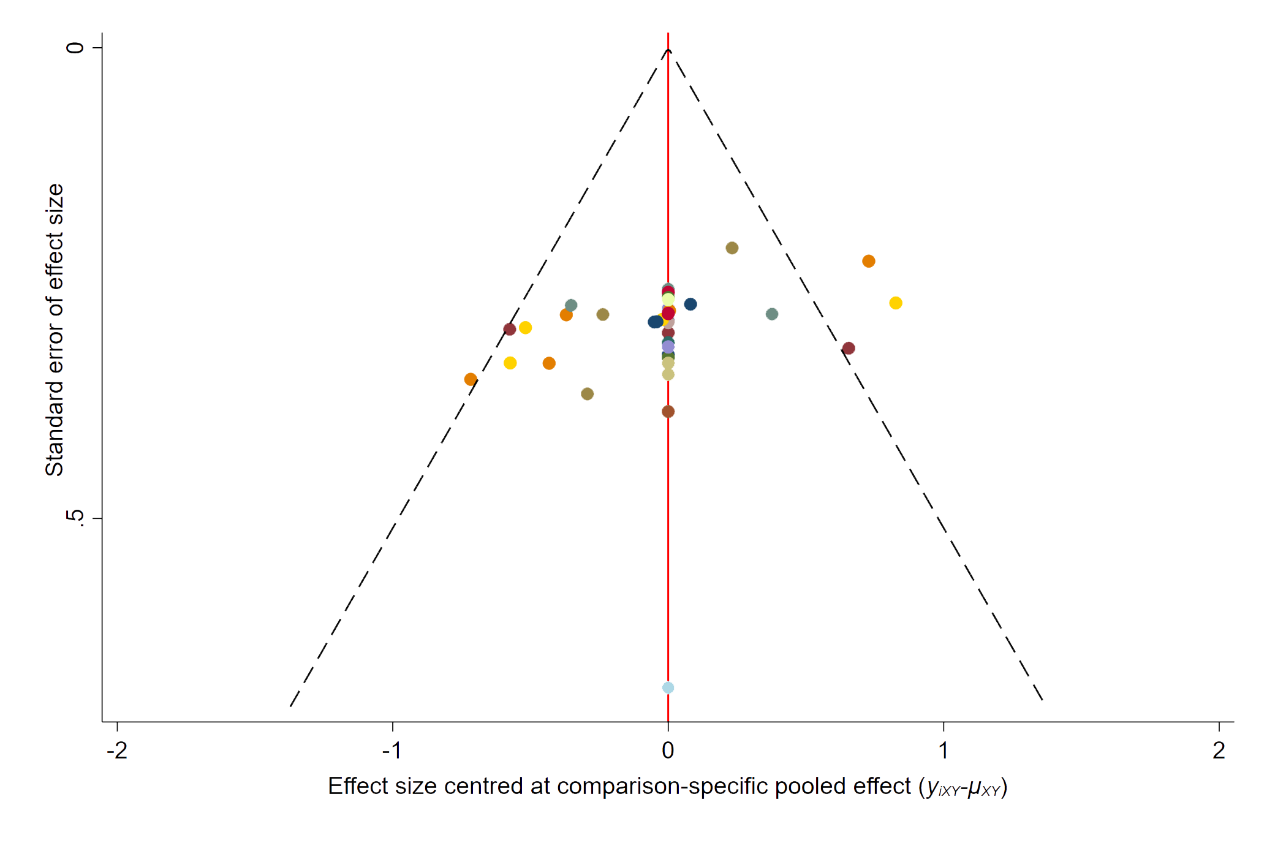
**

Figure 2: Comparison-adjusted funnel plot of all studies for network meta-analysis.

**Appendix 3**

**SUCRA and cumulative probability plots for**

**efficacy and acceptability**

**
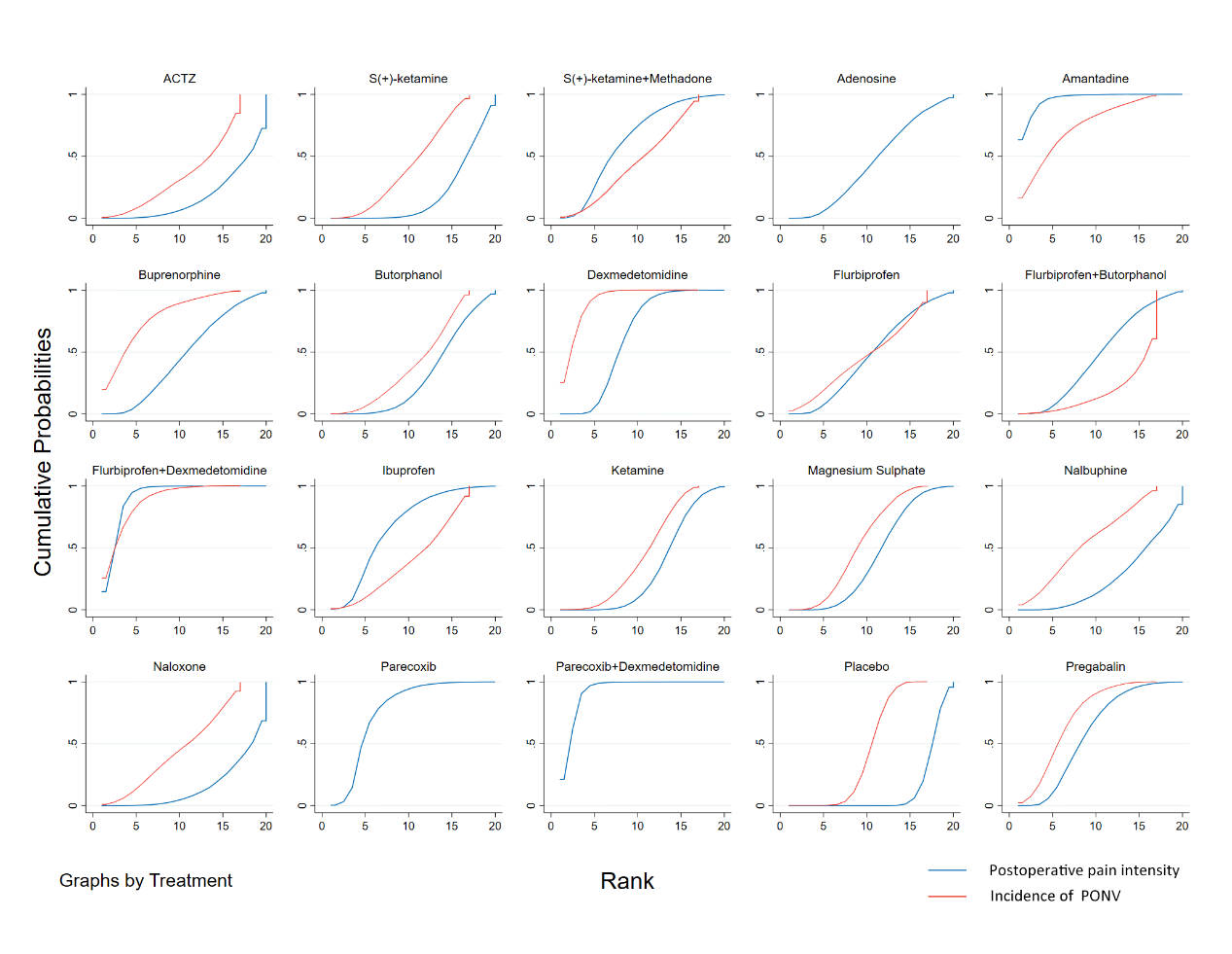
**

Figure 3: SUCRA and cumulative probability plots of network meta-analysis for postoperative pain intensity at rest at 24h and the incidence of PONV

**Appendix 4**

**Results of secondary outcomes**

**Appendix 4.1.**

**Pain thresholds at 24h after surgery**

**
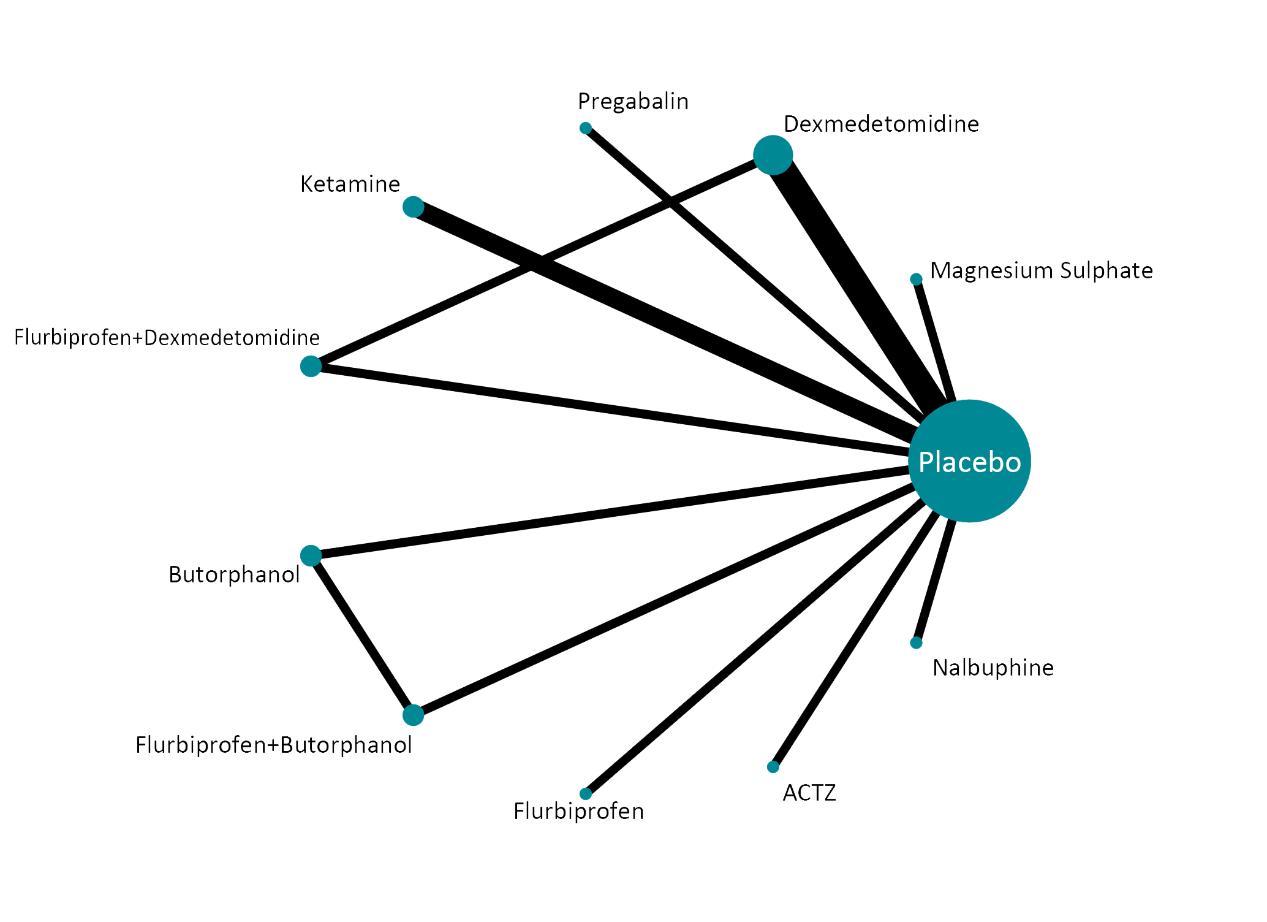
**

Figure 4.1.1: Network geometry plot of network meta-analysis for pain thresholds at 24h after surgery


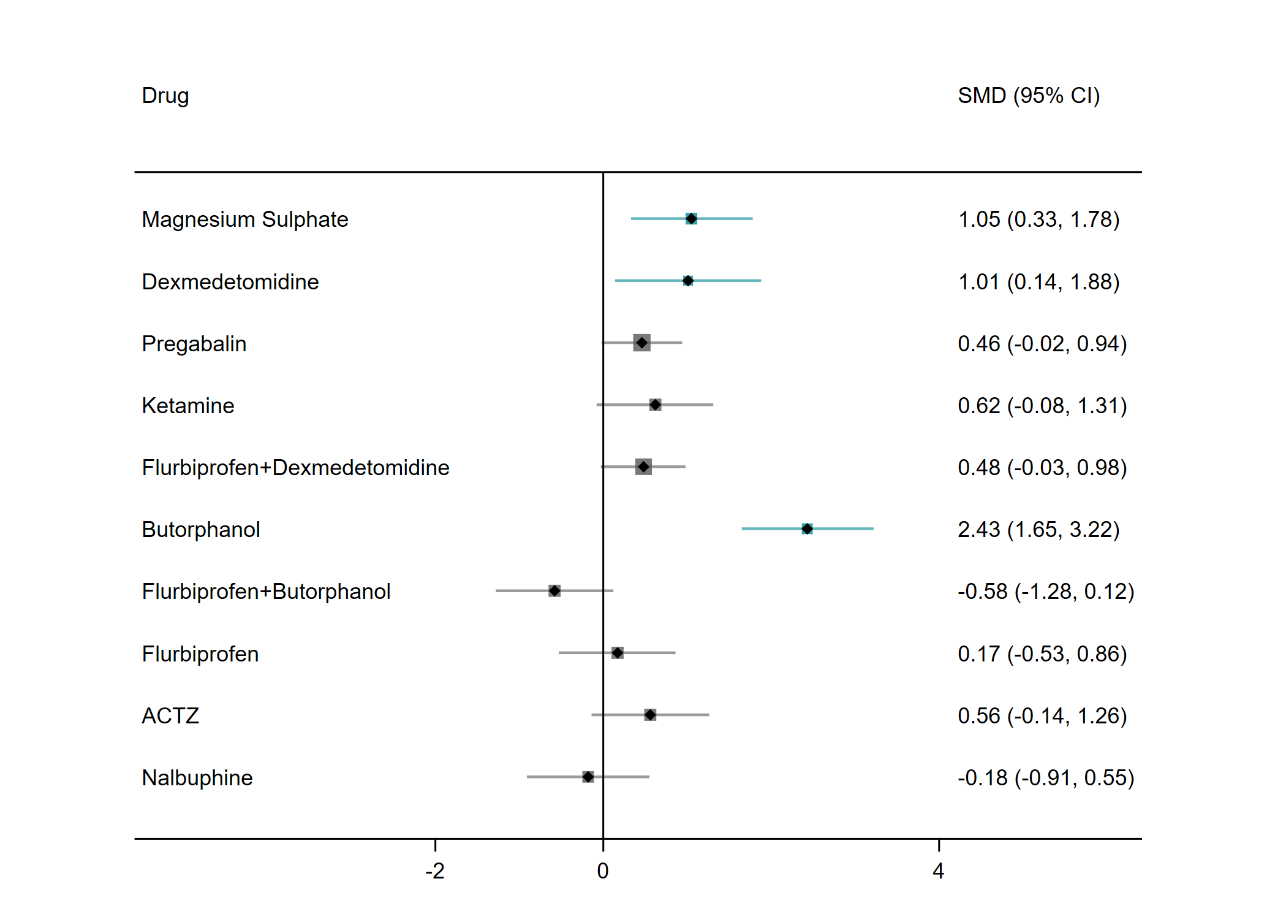
 Figure 4.1.2: Forest plots of network meta-analysis for pain thresholds at 24h after surgery

**
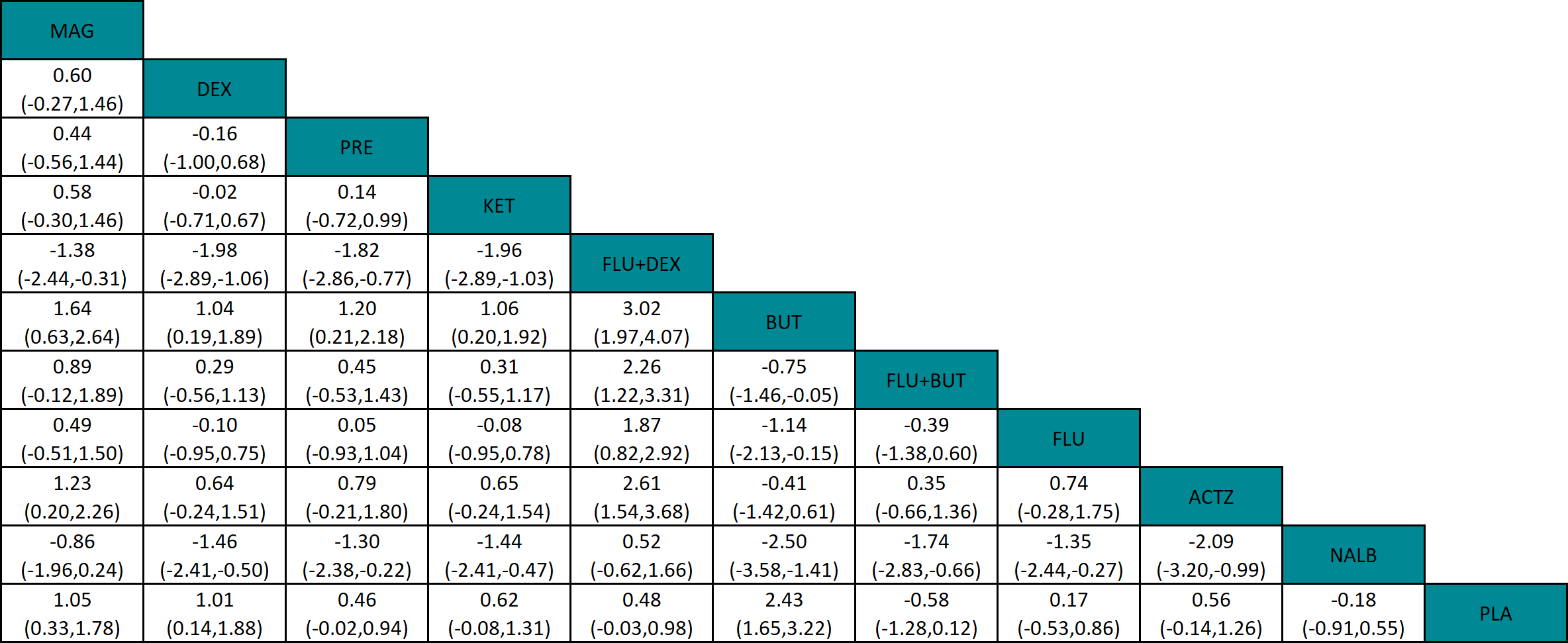
**

Figure 4.1.3: League table of network meta-analysis for pain thresholds at 24h after surgery

**
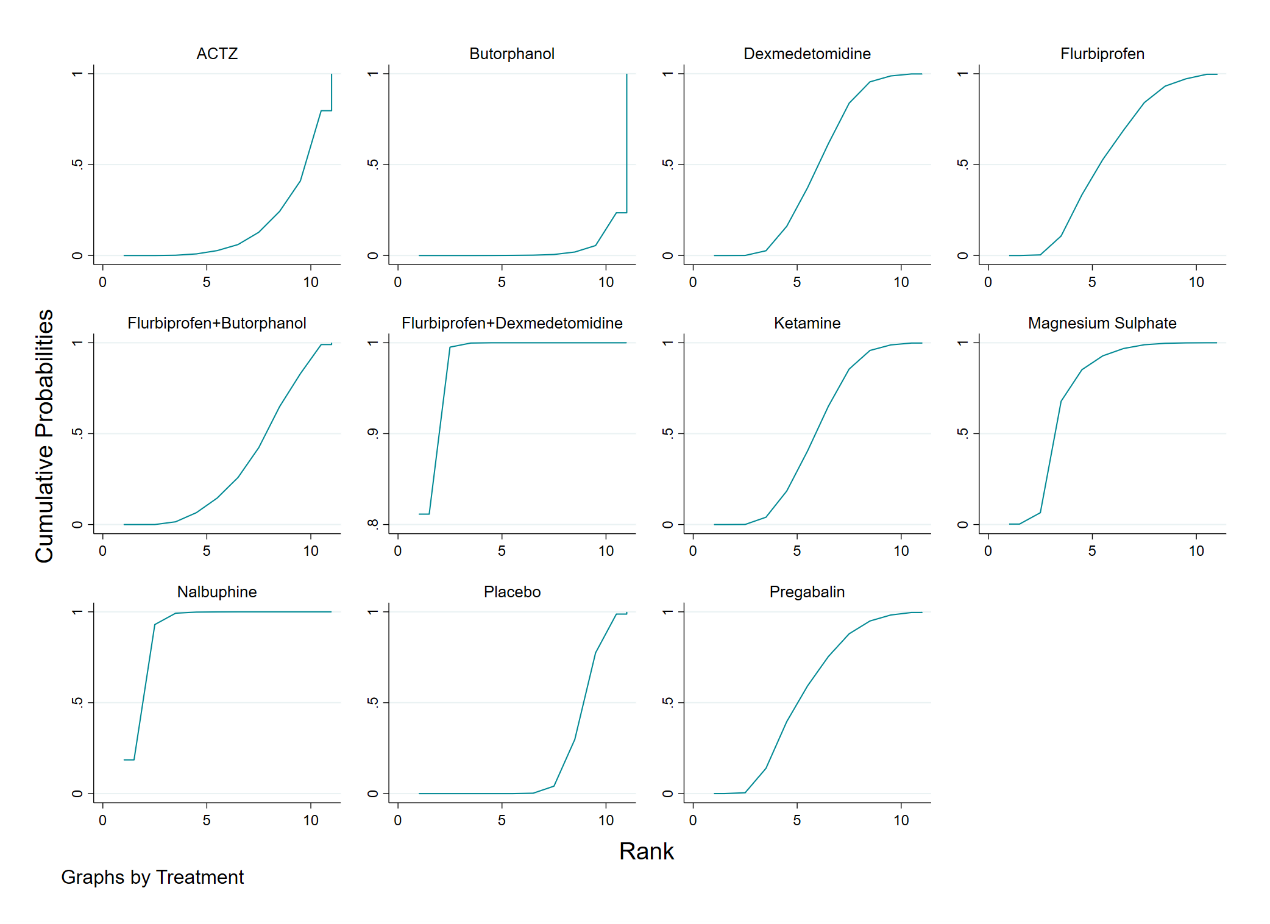
**

Figure 4.1.4: SUCRA and cumulative probability plots of network meta-analysis for pain thresholds at 24h after surgery

**Appendix 4.2**

**Cumulative morphine consumption over the 24h**

**
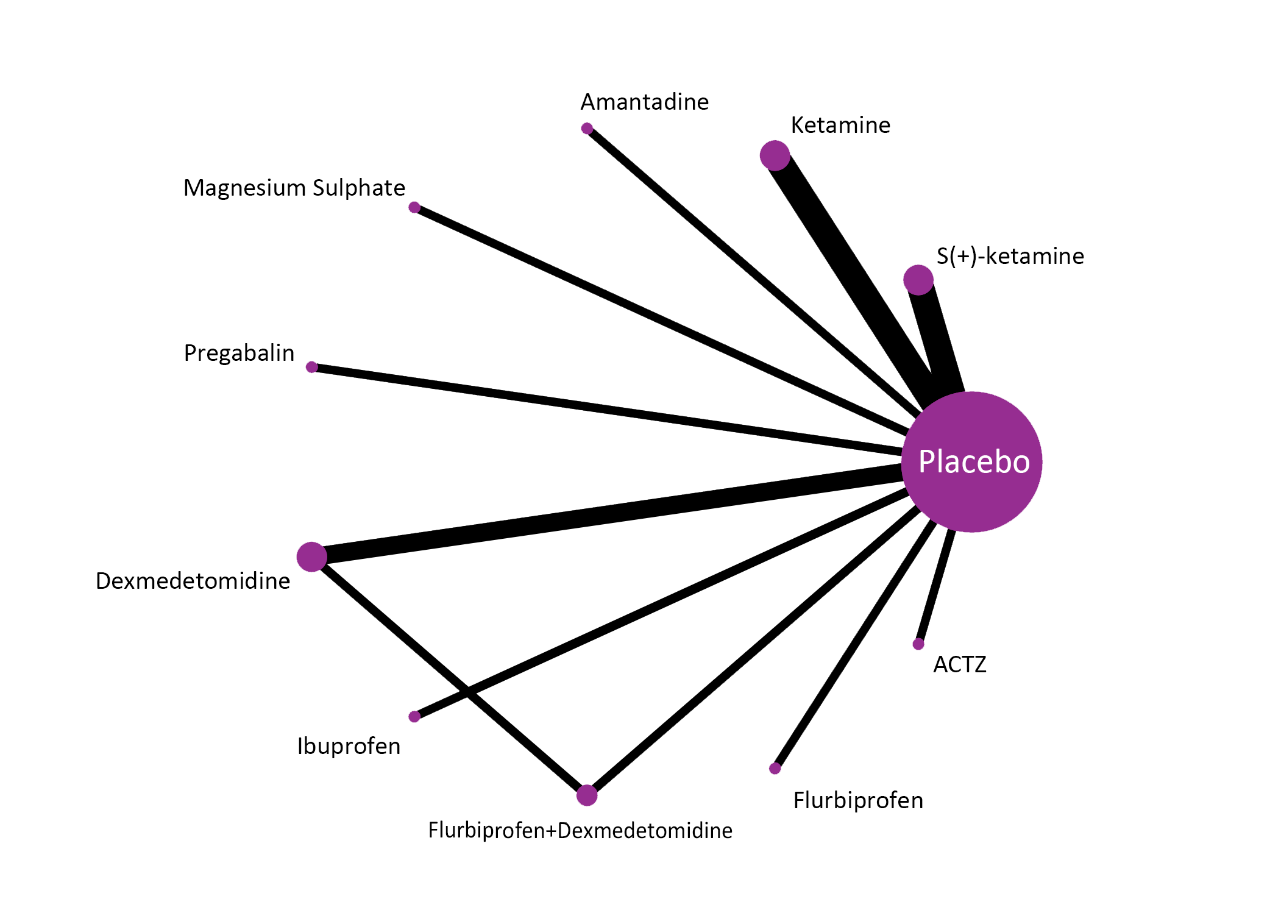
**

Figure 4.2.1: Network geometry plot of network meta-analysis for cumulative morphine consumption over the 24h


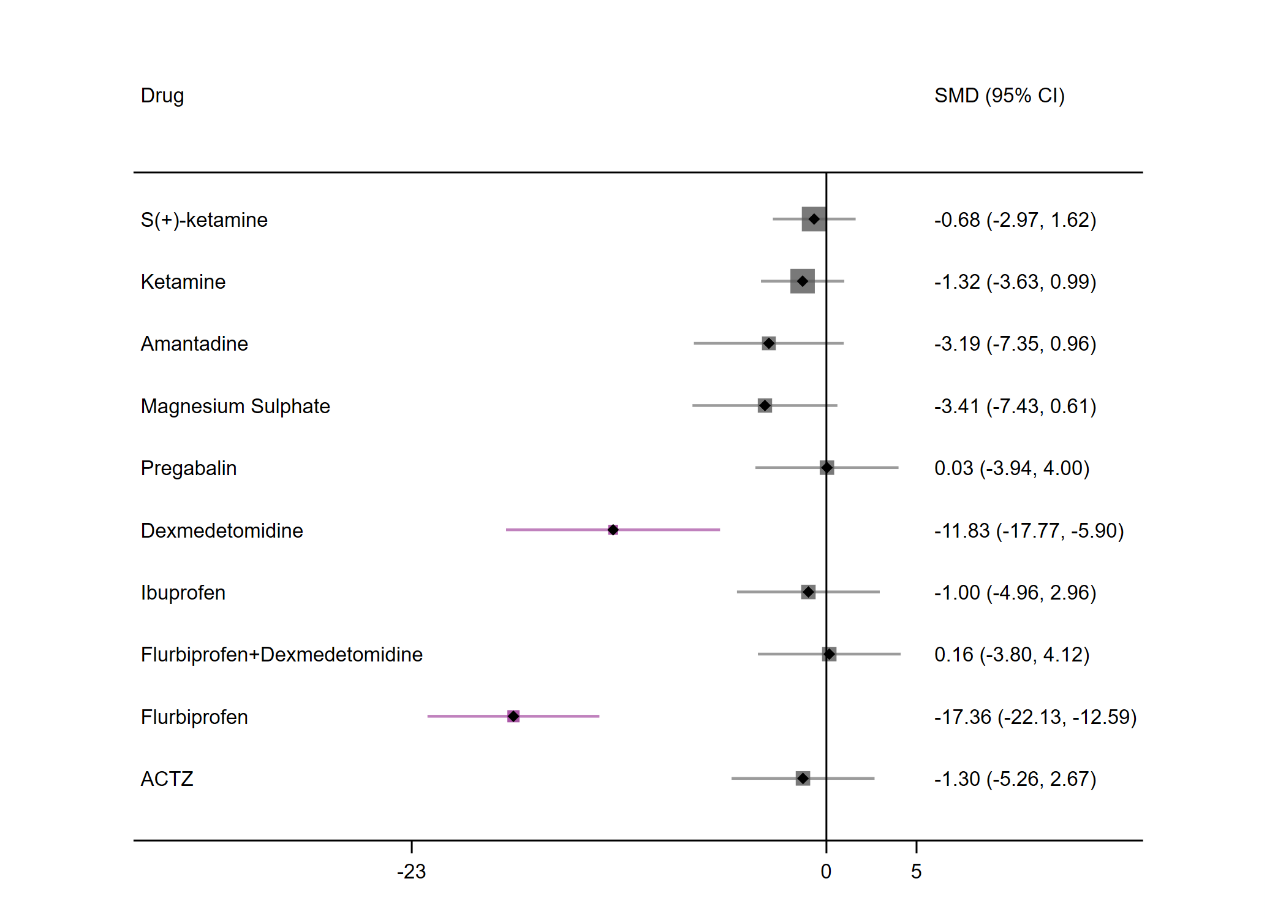
 Figure 4.2.2: Forest plots of network meta-analysis for cumulative morphine consumption over the 24h

**
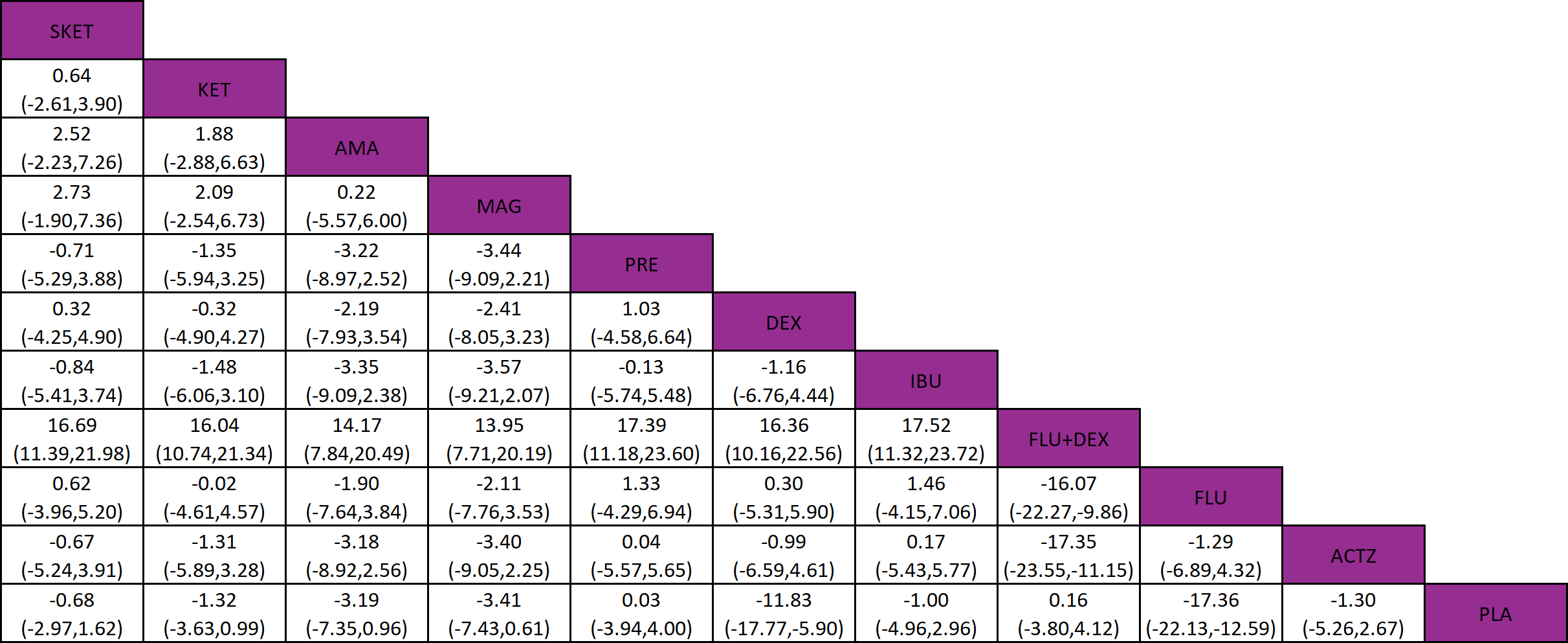
**

Figure 4.2.4: League table of network meta-analysis for cumulative morphine consumption over the 24h

**
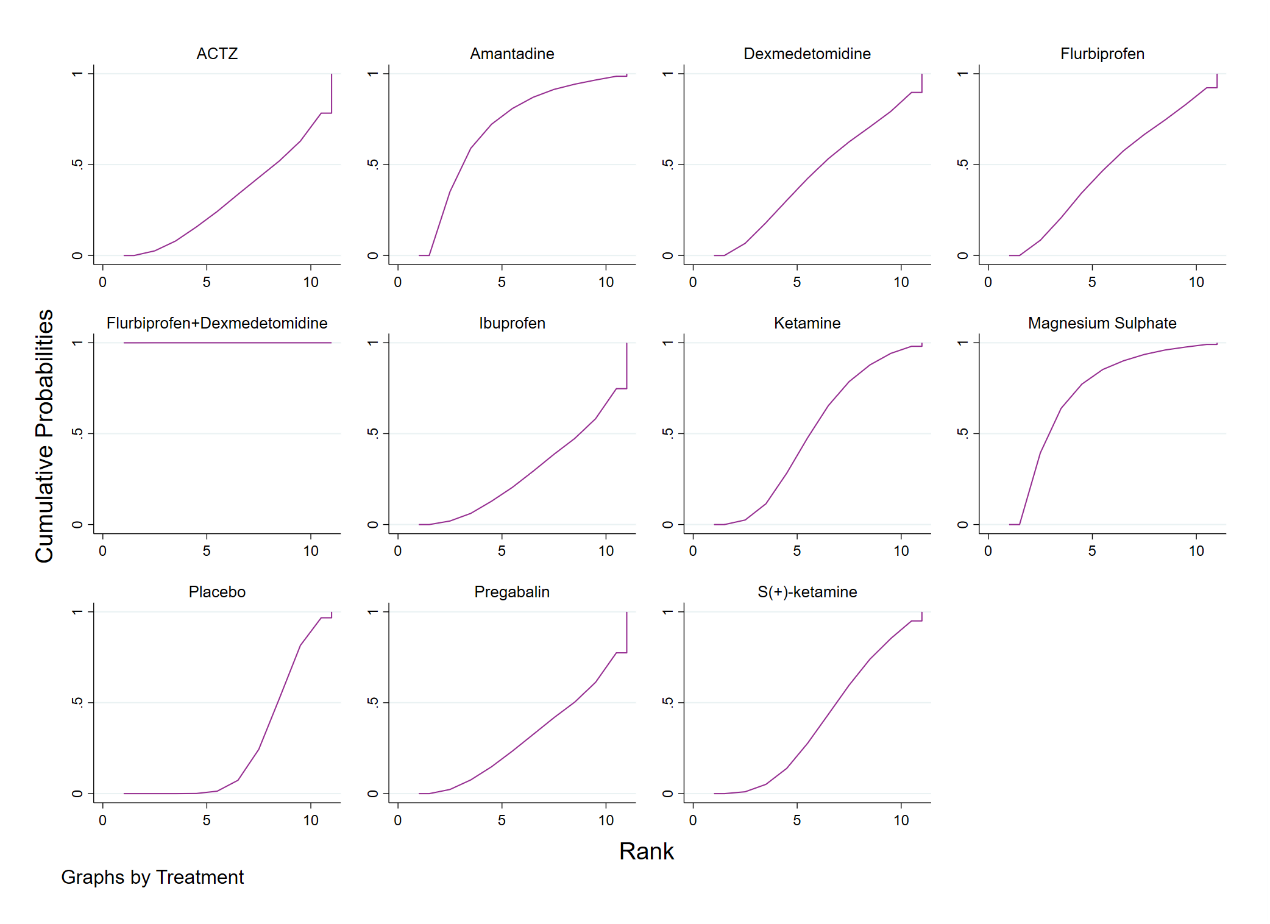
**

Figure 4.2.4: SUCRA and cumulative probability plots of network meta-analysis for cumulative morphine consumption over the 24h

**Appendix 4.3**

**The time to first postoperative analgesic requirement**

**
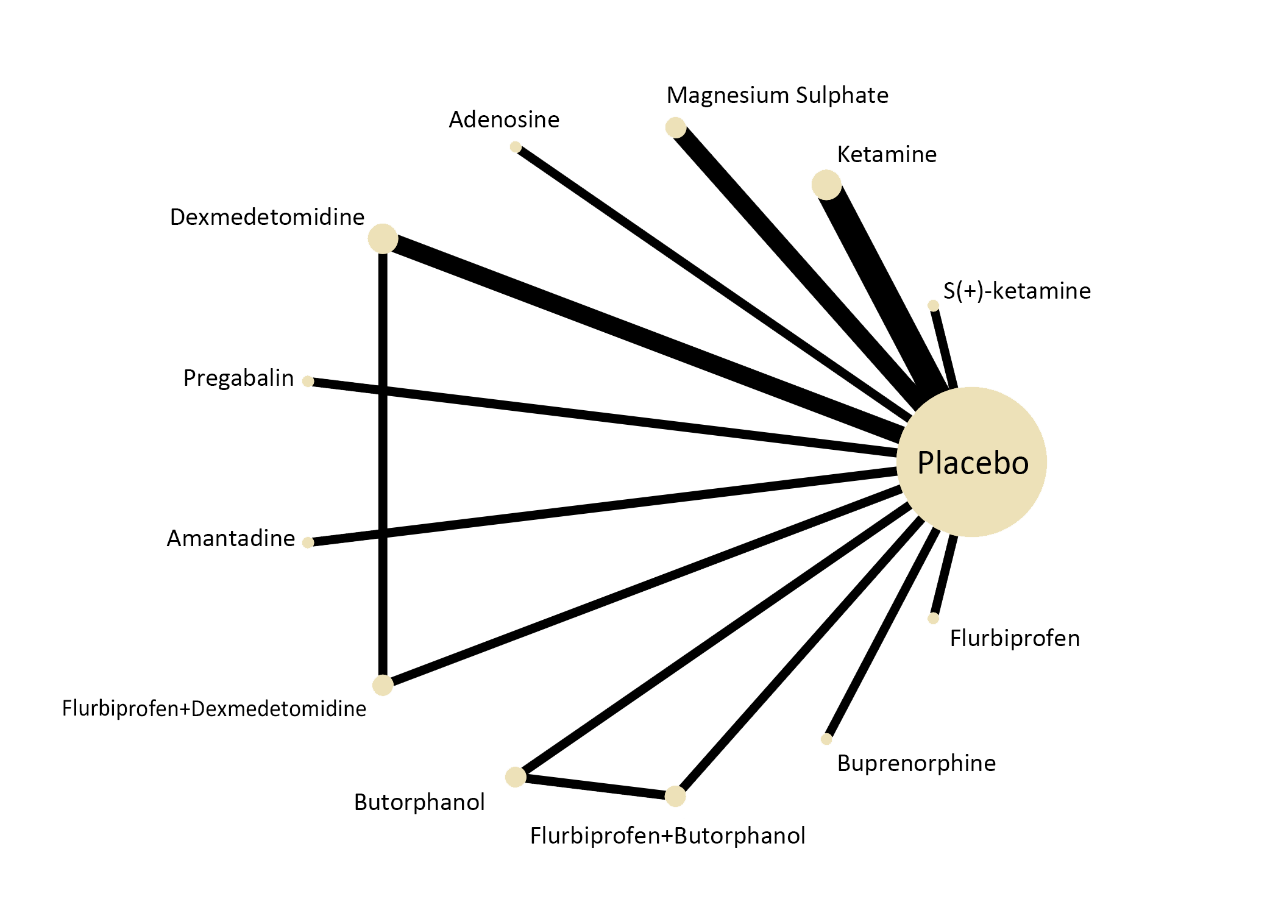
**

Figure 4.3.1: Network geometry plot of network meta-analysis for the time to first postoperative analgesic requirement


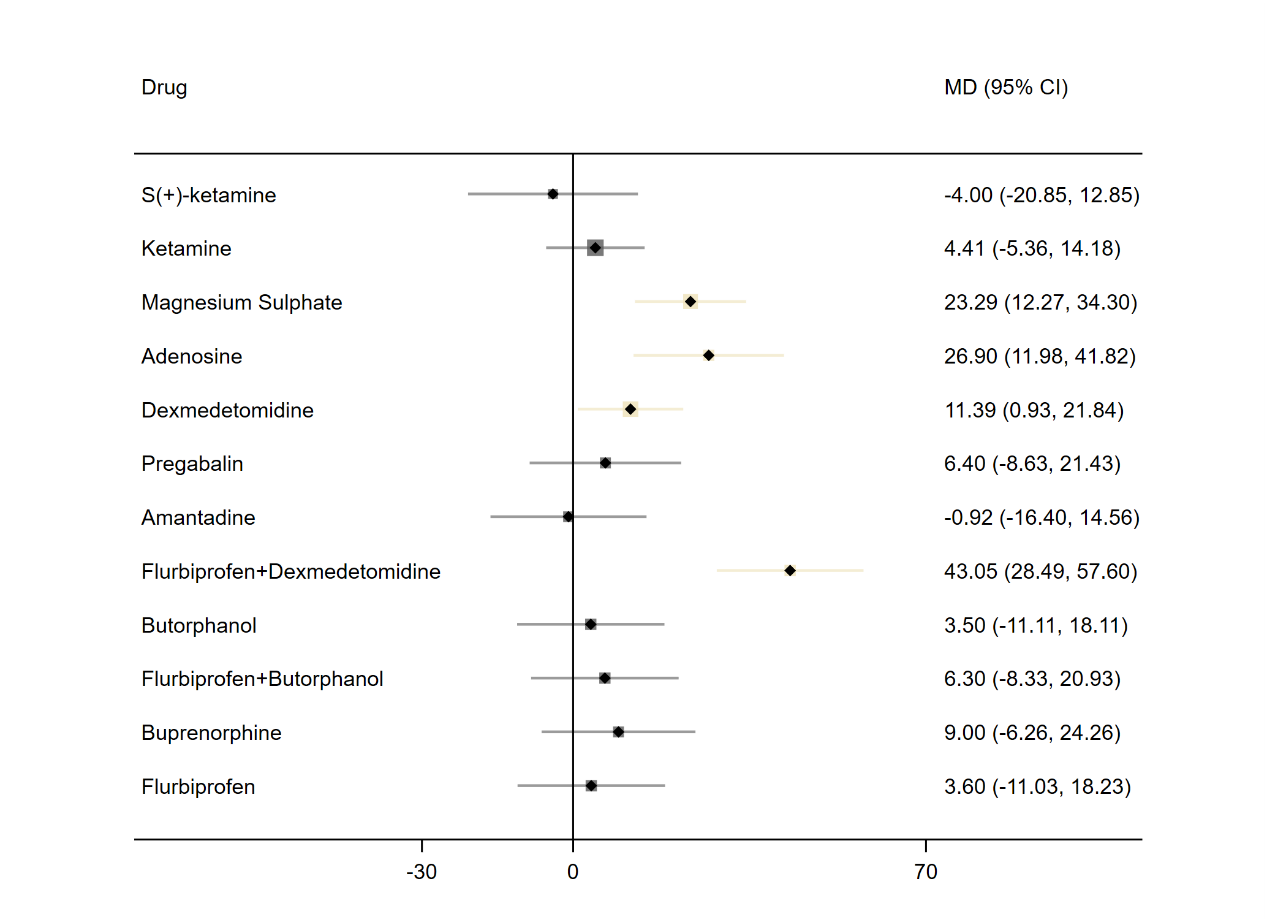
 Figure 4.3.2: Forest plots of network meta-analysis for the time to first postoperative analgesic requirement

**
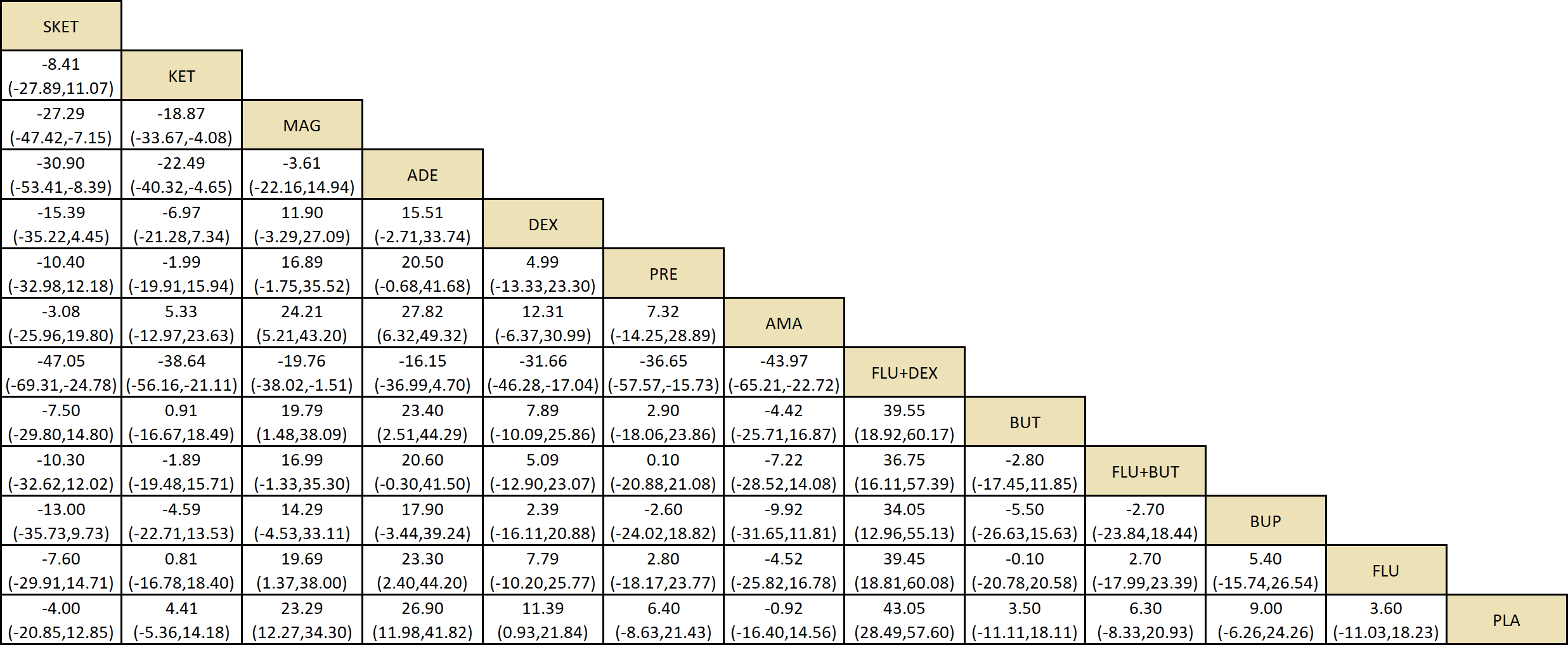
**

Figure 4.3.5: League table of network meta-analysis for the time to first postoperative analgesic requirement

**
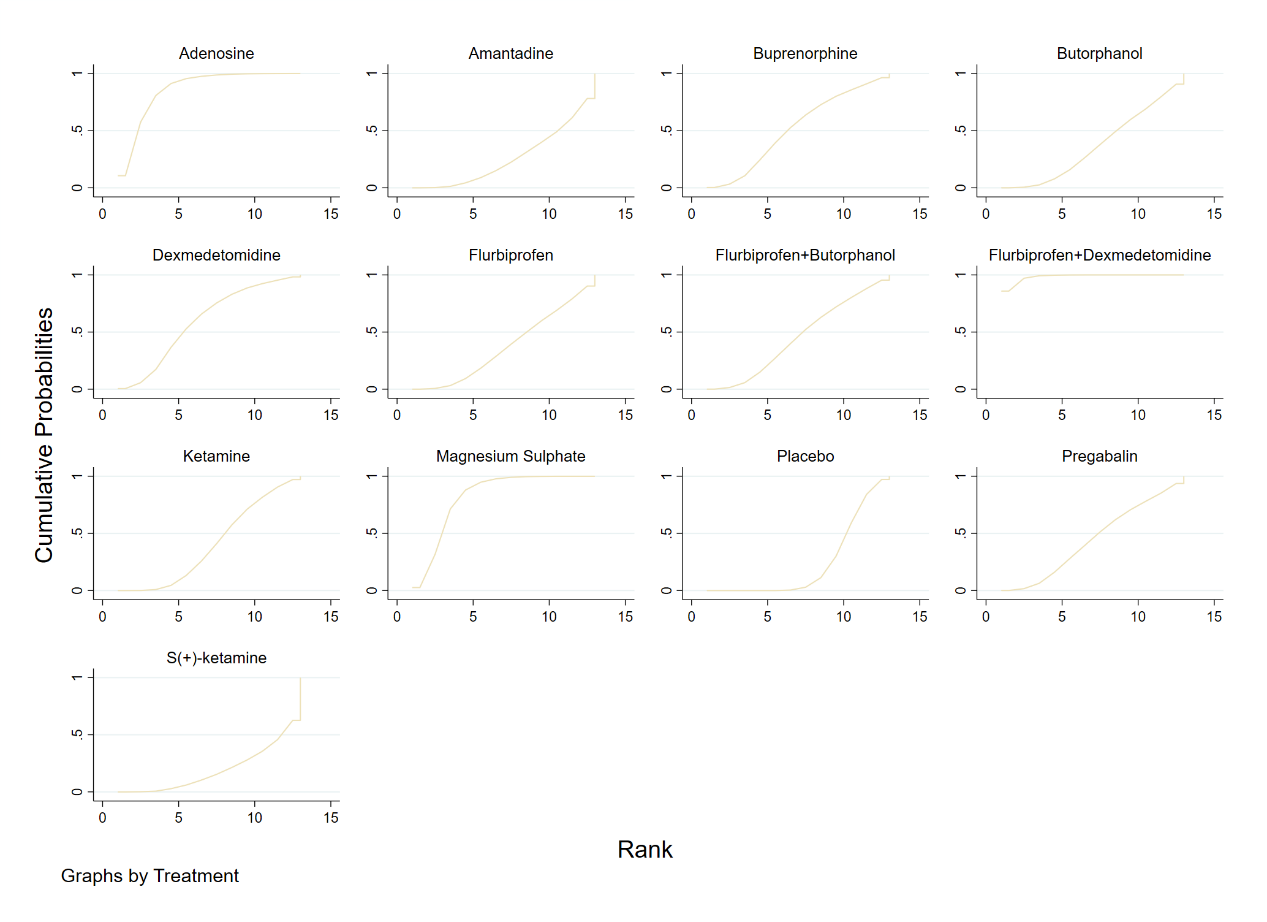
**

Figure 4.3.4: SUCRA and cumulative probability plots of network meta-analysis for the time to first postoperative analgesic requirement

**Appendix 4.4**

**Incidence of Shivering**

**
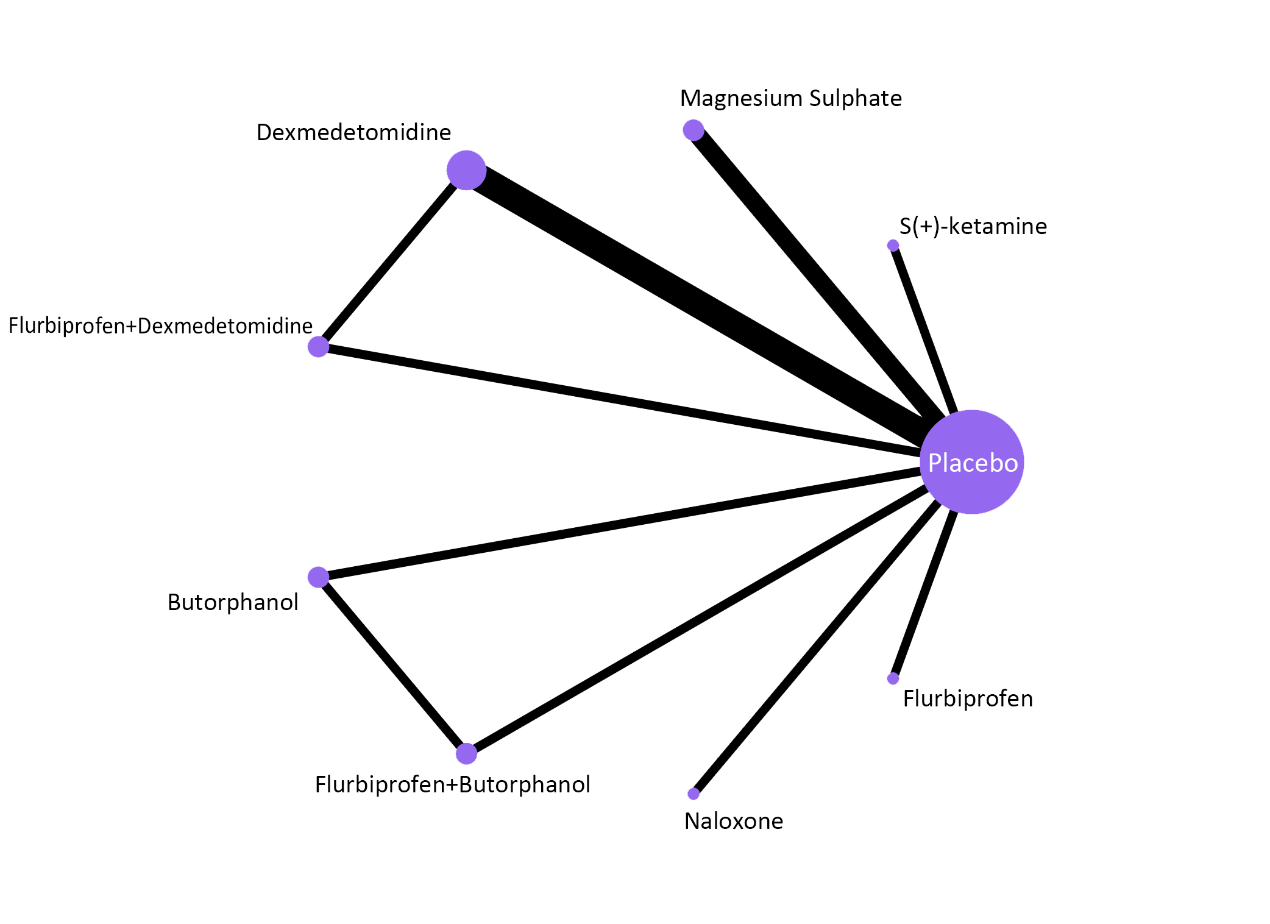
**

Figure 4.4.1: Network geometry plot of network meta-analysis for incidence of shivering


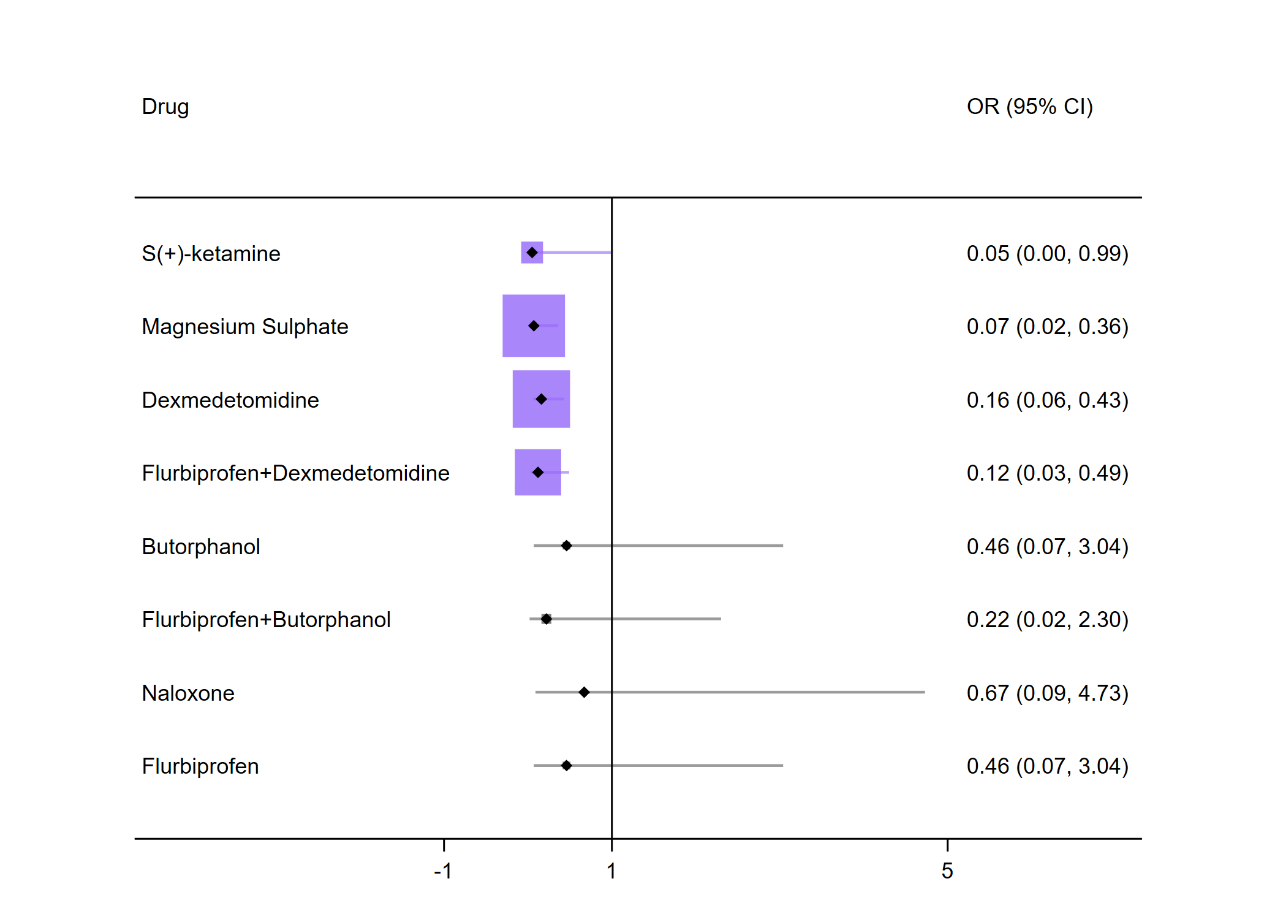
 Figure 4.4.2: Forest plots of network meta-analysis for incidence of shivering

**
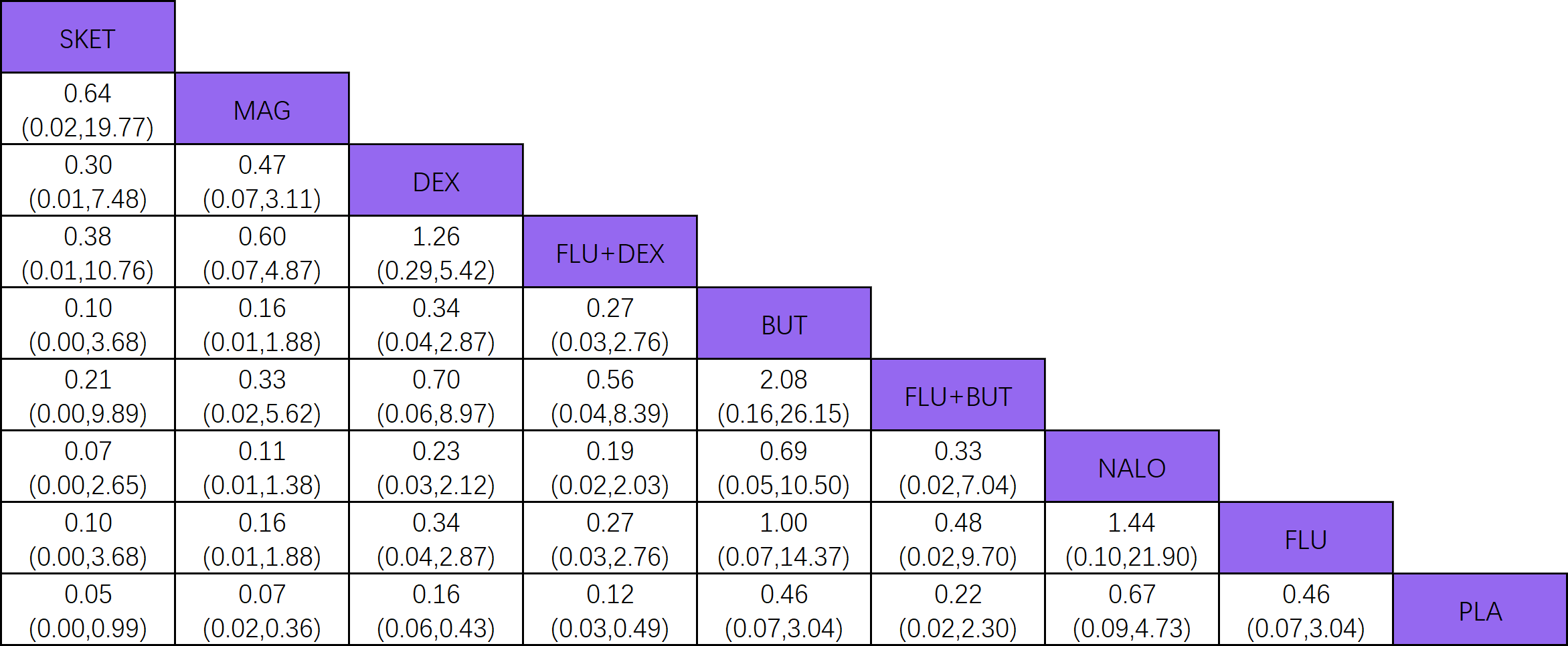
**

Figure 4.4.6: League table of network meta-analysis for incidence of shivering

**
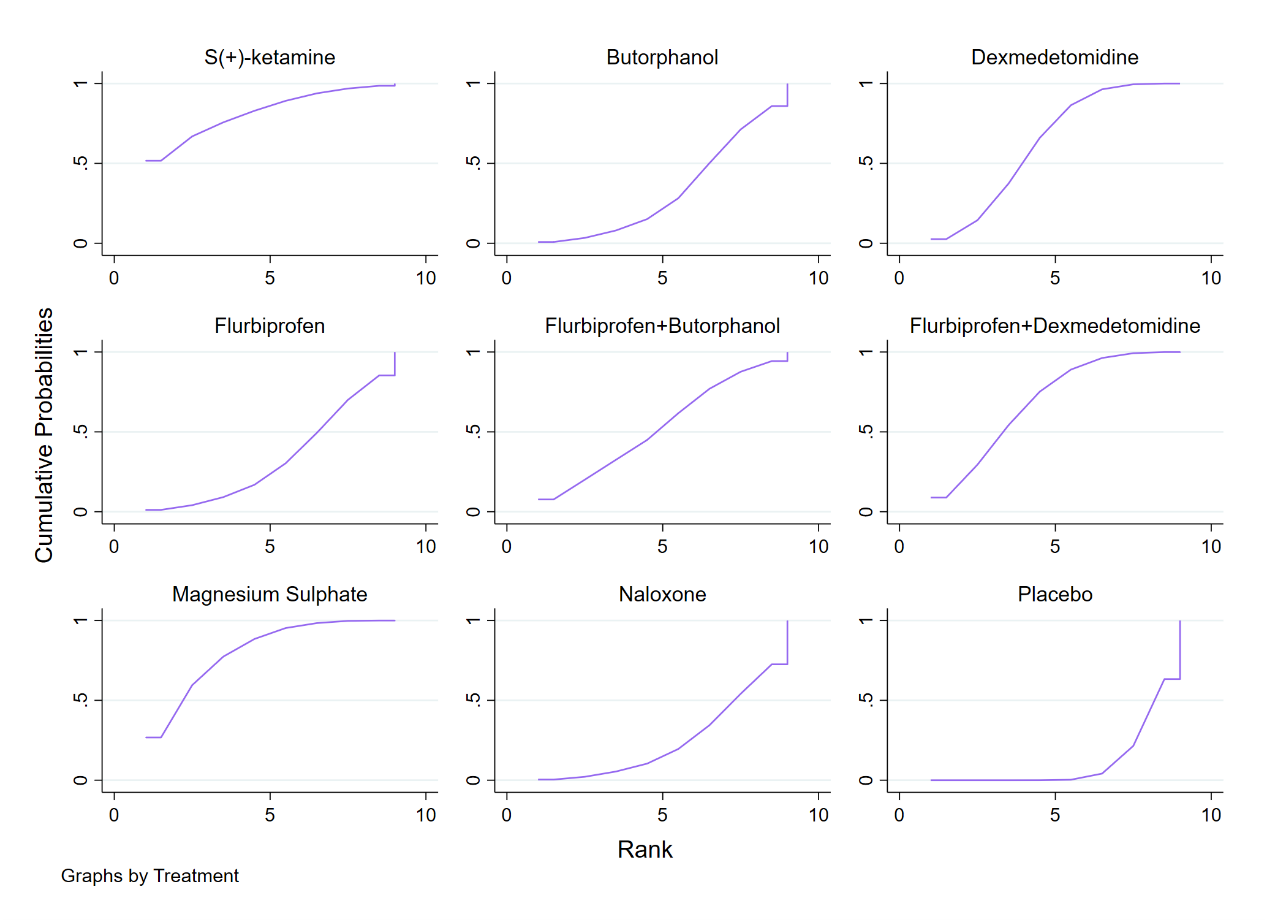
**

Figure 4.4.4: SUCRA and cumulative probability plots of network meta-analysis for incidence of shivering
